# Drug-resistant cancer cell-derived exosomal EphA2 promotes breast cancer metastasis via the EphA2-Ephrin A1 reverse signaling

**DOI:** 10.1038/s41419-021-03692-x

**Published:** 2021-04-20

**Authors:** Zicong Gao, Xingxing Han, Yuying Zhu, He Zhang, Ran Tian, Zhiyong Wang, Yanfen Cui, Zhaosong Wang, Ruifang Niu, Fei Zhang

**Affiliations:** 1 Public Laboratory, Tianjin Medical University Cancer Institute and Hospital, National Clinical Research Center for Cancer, Tianjin, 300060 China; 2 Key Laboratory of Cancer Prevention and Therapy, Tianjin, 300060 China; 3 Tianjin’s Clinical Research Center for Cancer, Tianjin, 300060 China; 4 Key Laboratory of Breast Cancer Prevention and Therapy, Tianjin Medical University, Ministry of Education, Tianjin, 300060 China

**Keywords:** Breast cancer, Metastasis

## Abstract

Tumor metastasis induced by drug resistance is a major challenge in successful cancer treatment. Nevertheless, the mechanisms underlying the pro-invasive and metastatic ability of drug resistance remain elusive. Exosome-mediated intercellular communications between cancer cells and stromal cells in tumor microenvironment are required for cancer initiation and progression. Recent reports have shown that communications between cancer cells also promote tumor aggression. However, little attention has been regarded on this aspect. Herein, we demonstrated that drug-resistant cell-derived exosomes promoted the invasion of sensitive breast cancer cells. Quantitative proteomic analysis showed that EphA2 was rich in exosomes from drug-resistant cells. Exosomal EphA2 conferred the invasive/metastatic phenotype transfer from drug-resistant cells to sensitive cells. Moreover, exosomal EphA2 activated ERK1/2 signaling through the ligand Ephrin A1-dependent reverse pathway rather than the forward pathway, thereby promoting breast cancer progression. Our findings indicate the key functional role of exosomal EphA2 in the transmission of aggressive phenotype between cancer cells that do not rely on direct cell–cell contact. Our study also suggests that the increase of EphA2 in drug-resistant cell-derived exosomes may be an important mechanism of chemotherapy/drug resistance-induced breast cancer progression.

## Introduction

Anthracyclines (doxorubicin, epirubicin) and taxanes (paclitaxel, docetaxel)-based chemotherapy regimens are very commonly used in the treatment of malignant tumors^1–3^. Despite high initial efficacy on most types of breast cancer, chemotherapy eventually fails, particularly for patients with advanced breast cancer^4–6^. The failure of chemotherapy is accompanied by the emergence of drug resistance and tumor relapse^7–9^. Recent studies have shown that chemotherapy can induce invasiveness and metastasis of breast cancer cells^10–12^. In addition, drug-resistant cancer cells always show an enhanced aggressive phenotype than their parental cells^13–16^. Collectively, under the stress of chemotherapeutic drugs, certain tumor cells have evolved additional abilities in addition to drug resistance, such as stronger invasion capabilities^17–22^. This phenomenon may be one of the causes of the rapid relapse of cancer patients after treatment failure. Thus, clarifying the molecular mechanisms underlying the pro-invasive and metastatic ability of certain chemotherapy is necessary.

Intercellular communications in tumor microenvironment are required for cancer initiation and progression^23–26^. Tumor cells can transmit or exchange messages with surrounding cells to promote cell proliferation, resistance to drugs, migration, and metastasis to distant organs. Extracellular vesicles, particularly exosomes, have been identified as the important carriers that transmit specific substances to neighboring or distant cells^27–29^. Exosome-mediated intercellular communications between cancer cells and stromal cells are essential for tumor growth, angiogenesis, drug resistance, immune escape, and metastasis^28,30,31^. Recently, two studies have suggested that gemcitabine-treated pancreatic cancer (PC) cells release exosomes to increase the chemoresistance of sensitive PC cells^32,33^. These findings indicate that exosome-mediated communications between cancer cells also contribute to cancer progression. However, little attention has been concerned to this aspect.

EphA2 belongs to the Eph kinase family, the largest subfamily of receptor tyrosine kinase superfamily. The prominent ligand of EphA2 is Ephrin A1, which is anchored to the cell surface via a glycosylphosphatidylinositol moiety^34,35^. Hence, the binding of EphA2 to Ephrin A1 on a neighboring cell depends on cell–cell contacts and leads to bidirectional signals in the corresponding cells^35^. The forward signal is transmitted in EphA2-expressing cells, whereas the reverse signal is transmitted in Ephrin A1-expressing cells. The Eph-Ephrin system constitutes an important intercellular communication system and plays a fundamental role in the normal physiology and pathogenesis of many diseases, including cancer^36–40^. Deregulated EphA2/Ephrin A1 signal is observed in many types of tumors, particularly breast cancer. The elevated expression of EphA2 is correlated with tumor deterioration and poor prognosis of cancer patients^39^. Nevertheless, the detailed mechanism, through which EphA2 contributes to breast cancer progression, remains largely unknown.

Although previous studies have considered that EphA2/Ephrin A1 signal transduction occurs at the cell–cell junction that requires direct cell–cell contact, recent evidence has shown that the Eph receptors and ligands are also expressed on exosomes, indicating that exosomal Eph/Ephrin molecules can transmit long-range signals without direct cell–cell interaction^41–43^. However, whether exosomal EphA2 are involved in breast cancer progression remains unknown. In this study, we reported that the exosomes released by drug-resistant breast cancer cells were rich in EphA2 protein. The exosomal EphA2-Ephrin A1 reverse pathway rather than the forward pathway confers the aggressive phenotype transfer from resistant cells to sensitive cells that does not require direct cell–cell contact. Moreover, the activation of ERK1/2 signaling downstream of the reverse pathway may be related to the promotion of the invasion and metastasis of breast cancer cells by exosomal EphA2. Collectively, our results indicate that the increase of exosomal EphA2 may be an important mechanism of chemotherapy/drug resistance-induced breast cancer progression.

## Results

### Exosomes derived from drug-resistant cells enhance breast cancer cell migration and invasion

**Fig. 1:**
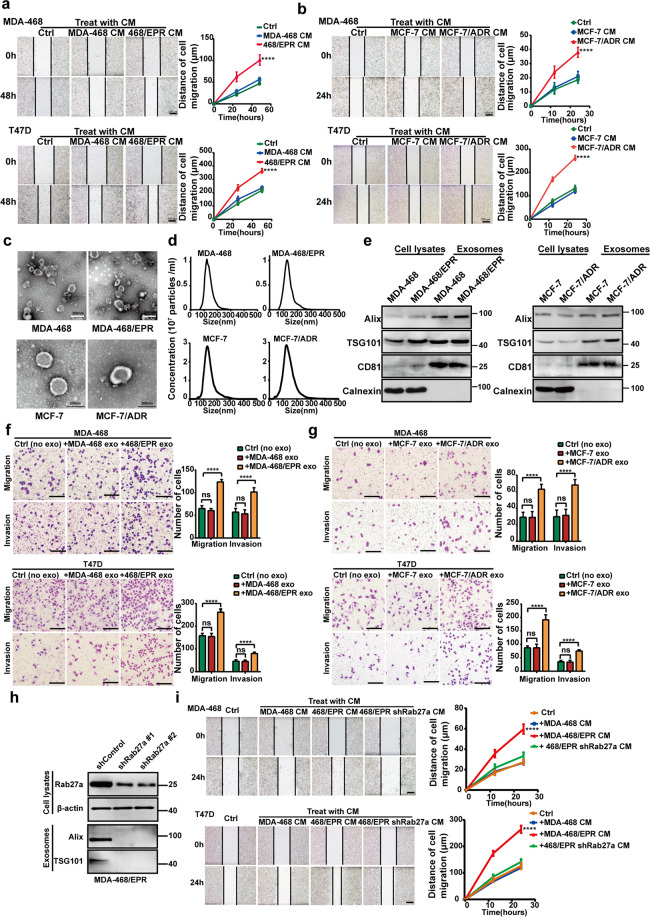
Exosomes derived from drug-resistant cells enhance breast cancer cell migration and invasion. **a**, **b** CM from drug-resistant cells significantly enhanced the migratory ability of human breast cancer MDA-MB-468 cells and T47D cells compared with CM from parental cells and fresh medium control as measured by wound healing assay. All experiments were repeated at least three times. ****P < 0.0001. Scale bars: 200 μm. **c** Transmission electron microscopic image of exosomes derived from drug-resistant cells and its parental cells. Scale bars: 200 nm. **d** Nanoparticle tracking analysis (NTA) of exosomes derived from drug-resistant cells and its parental cells. **e** Equal amounts of protein (100 μg) from drug-resistant cells and its parental cells and exosomes were analyzed by Western blotting. Alix, TSG101, and CD81 were used as the positive control of exosomes, and Calnexin was used as the negative control of exosomes. **f, g** Exosomes derived from drug-resistant cells (DR-Exos) significantly increased the migration and invasion ability of two breast cancer cells compared with exosomes derived from drug-sensitive cells (DS-Exos). For cell migration assay, 5 × 10^4^ cells suspended in 200 μL of serum-free medium were loaded onto the upper chambers. Six hundred microliters of medium with 10% FBS was added into the lower chamber. For cell invasion assay, 1 × 10^5^ cells suspended in 200 μL of serum-free medium were loaded onto the upper chambers coated with Matrigel. The incubation time was 24 h. The statistical results were summarized in the right panel. Data were expressed as mean ± SD. All experiments were repeated at least three times. ****P < 0.0001 and ^ns^*P* > 0.05 indicate no statistical significance. Scale bars: 200 μm. **h** Knockdown of Rab27a reduced the amounts of exosomes derived from MDA-MB-468/EPR cells. **i** Knockdown of Rab27a suppressed the migration-promoting effect of CM derived from drug-resistant cells. All experiments were repeated at least three times. ****P < 0.0001. Scale bars: 200 μm.

Tumor cell–cell communication promotes cancer progression in the tumor environment. The acquisition of drug resistance by cancer cells always evolves an enhanced invasive and metastatic phenotype. We hypothesized that this aggressive phenotype can be transmitted from drug-resistant cells to sensitive cells. To investigate this possibility, we made conditioned medium (CM) from drug-resistant cells (DR-CM) or its parental drug-sensitive cells (DS-CM) and then treated breast cancer cells with CM. As shown in Fig. 1a, b, wound healing assay showed that DR-CM significantly enhanced the migratory ability of two human breast cancer cells compared with DS-CM and the fresh medium control. Exosomes are emerging as a central role in cell–cell communication. To investigate whether exosomes mediate this migration-promoting effect, we isolated exosomes from drug-resistant cells and their parental cells. The structural features of exosomes were confirmed by TEM and nanoparticle tracking analysis. As shown in Fig. 1c, d, the diameter distribution of the purified exosomes ranged from 30 to 200 nm. In addition, the isolated exosomes were rich in exosomal specific markers, and the absence of Calnexin indicated that the exosomes were not contaminated by cytoplasmic content (Fig. 1e). Next, cells expressing GFP were incubated with PKH-26-labeled exosomes. The results showed that the stained exosomes could be endocytosed into the recipient cells (Supplementary Fig. 1a, b). As shown in Fig. 1f, g, exosomes derived from drug-resistant cells (DR-Exos) significantly increased the migration and invasion ability of two breast cancer cells compared with exosomes derived from drug-sensitive cells (DS-Exos). By contrast, the migration-promoting effect of DR-CM was suppressed by the knockdown of Rab27a, a GTPase that is essential for exosome secretion (Fig. 1h, i). Collectively, these results indicate that DR-Exos promote the migration and invasion of drug-sensitive breast cancer cells. This aggressive phenotype can be transmitted from drug-resistant cells to sensitive cells. It has been reported that drug pump P-glycoprotein (P-gp) can be transferred between drug-resistant and drug-sensitive human cancer cells via extracellular vesicles^44,45^. We also found that P-gp could transferred from MCF-7/ADR cells to T47D cells via exosomes (Supplementary Fig. 2a, b).

### EphA2 protein is enriched in exosomes derived from drug-resistant cells

**Fig. 2:**
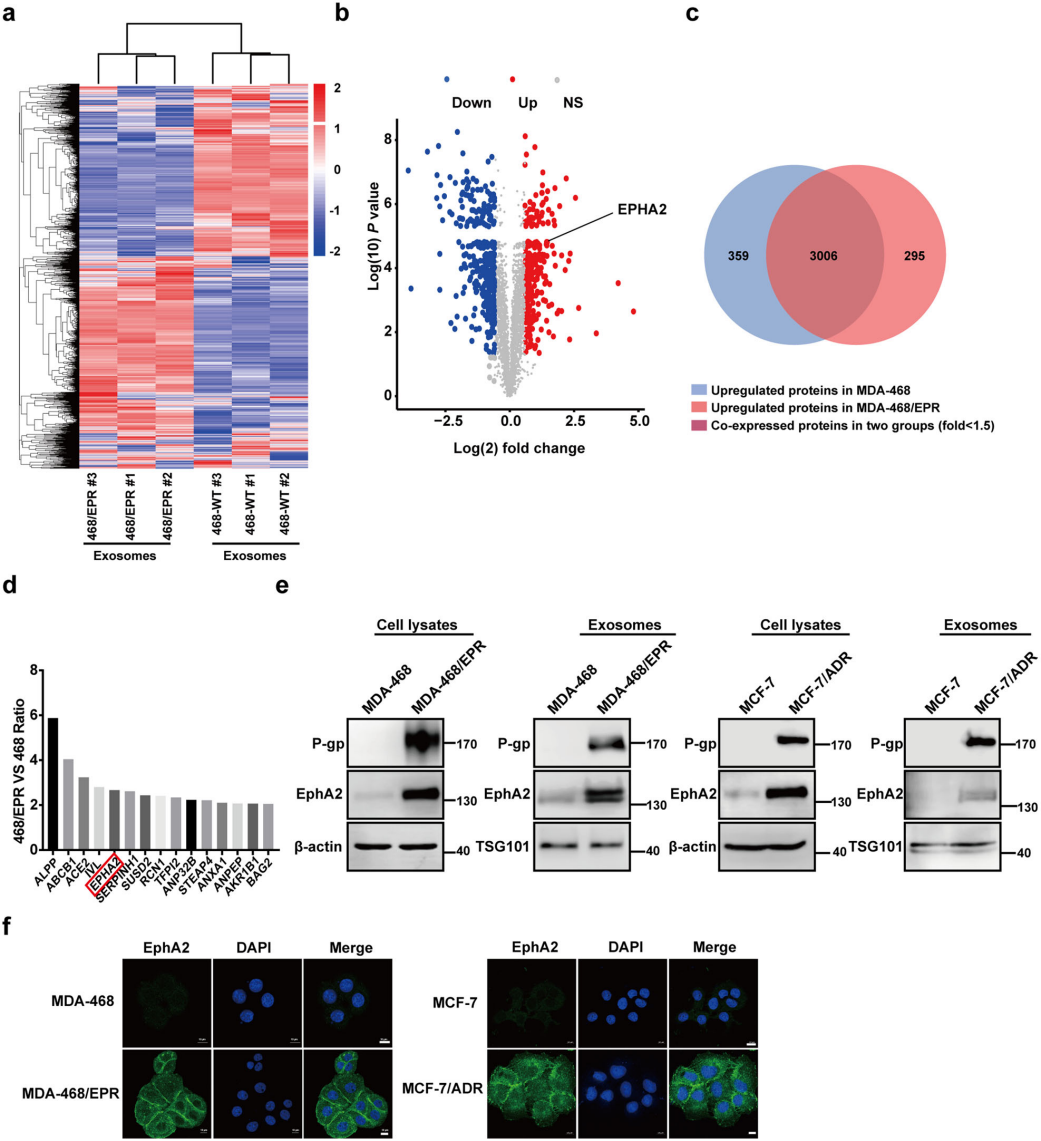
EphA2 protein is enriched in exosomes derived from drug-resistant cells. **a** Heat map of exosomal proteins differentially expressed in MDA-MB-468 and MDA-MB-468/EPR cells. Exosomes were isolated from drug-resistant breast cancer cells and their parental cells, and then a TMT-based quantitative proteomic method was performed to identify differentially expressed proteins in two kinds of exosomes. **b** Volcano map of exosomal proteins differentially expressed in MDA-MB-468 and MDA-MB-468/EPR cells. Blue and red dots represent the proteins significantly upregulated in exosomes from MDA-MB-468 and MDA-MB-468/EPR cells. **c** The Venn diagram of different exosomal proteins in MDA-MB-468 cells and MDA-MB-468/EPR cells. **d** Fifteen proteins were identified by using a strict cutoff (fold change >2.00, score >100). **e** The expression of EphA2, ABCB1 (encode P-glycoprotein) in exosomes, and cell lysates were analyzed by using Western blotting; β-actin was used as the loading control. **f** The expression of EphA2 in two drug-resistant breast cancer cells and their parental cells was studied by using immunofluorescence staining. Scale bars: 10 μm.

We performed comparative proteomics analysis of exosomes derived from drug-resistant MD-MB-468/EPR and parental cells by mass spectrometry to explore the underlying mechanisms. In general, 3660 unique proteins were identified and quantified in both samples. Several representative MS/MS spectra are shown in Supplementary Fig. 3a. A total of 295 proteins were upregulated, and 359 proteins were downregulated in the DR-Exos compared with DS-Exos (fold change > 1.5) (Fig. 2a–c). A strict cutoff was used (fold change > 2.00, score > 100) to screen for proteins with important biological significance in DR-Exos, and 15 proteins were identified (Fig. 2d). Among those proteins, Eph receptor tyrosine kinase EphA2, a well-known metastatic-promoter, was selected and analyzed by using Western blotting to verify our proteomics data. Consistently, immunoblotting and immunofluorescence confirmed that the expression of EphA2 was higher in DR-Exos than that in DS-Exos (Fig. 2e, f). Moreover, the expression of ALPP, ABCB1, ACE2, IVL, SERPINH1, ANXA1, and ANPEP in DR-Exos was also significantly higher than DS-Exos (Supplementary Fig. 4), suggesting that our proteomics results are convincing.

### Exosomal EphA2 promotes migration and invasion of breast cancer cells

**Fig. 3:**
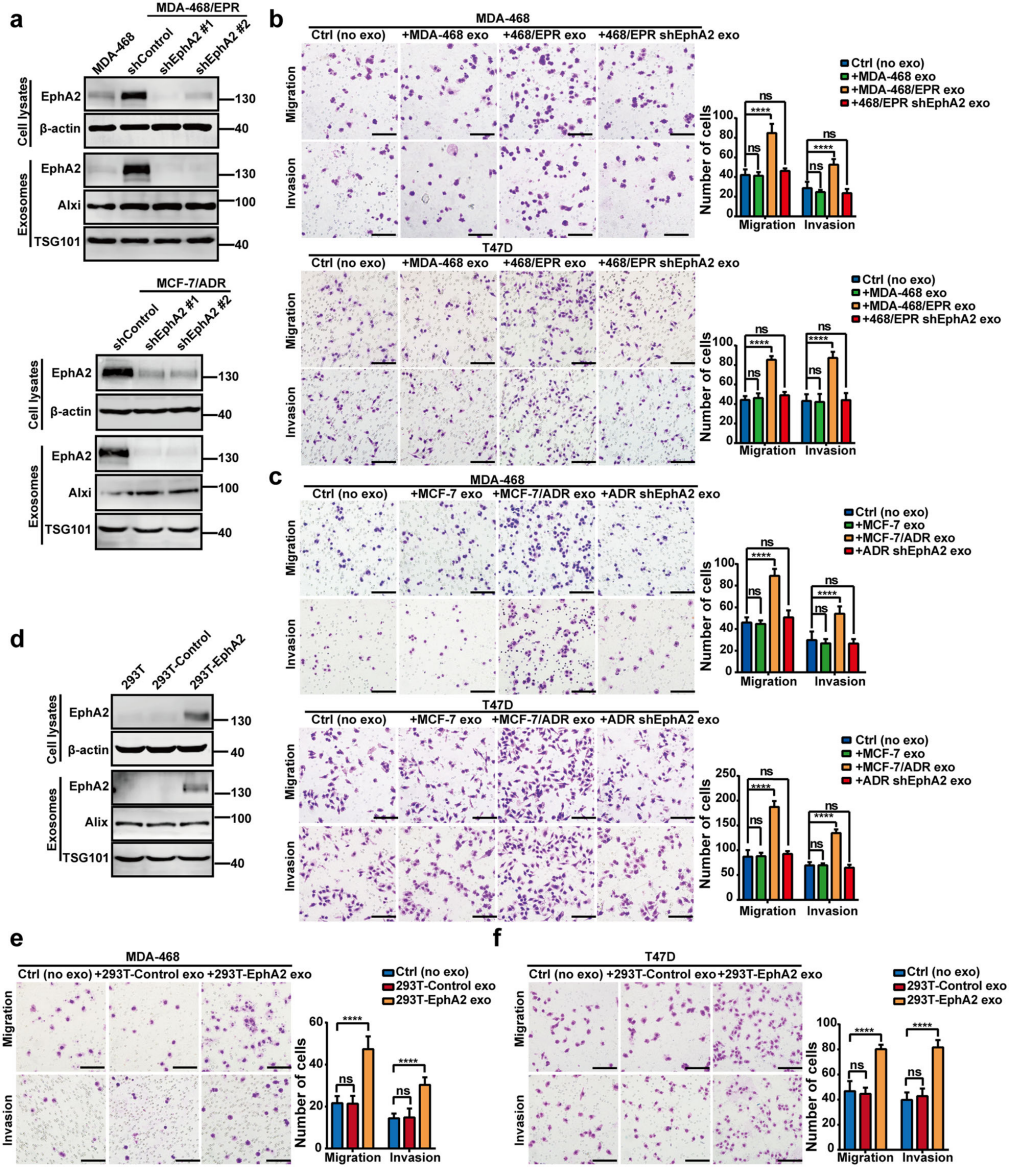
Exosomal EphA2 promotes the migration and invasion of breast cancer cells. **a** Western blotting analysis showed that the expression of EphA2 was silenced in drug-resistant cells and exosomes after infection with lentivirus expressing EphA2-specific shRNAs; β-actin was used as the loading control. **b**, **c** Exosomes from EphA2-silenced drug-resistant cells disabled the migratory and invasive promoting effect in breast cancer cells. All experiments were repeated at least three times. ****P < 0.0001 and ^ns^*P* > 0.05 indicate no statistical significance. **d** Western blotting analysis showed that the expression of EphA2 in HEK-293T cells and exosomes was transfected with control EphA2 vectors. β-actin was used as the loading control. **e, f** Exosomes from EphA2-expressing HEK-293T cells significantly promoted the migration and invasion ability of breast cancer cells compared with control exosomes. The statistical results were summarized in the right panel. All experiments were repeated at least three times. ****P < 0.0001 and ^ns^*P* > 0.05 indicate no statistical significance. Scale bars: 200 μm.

We silenced the expression of EphA2 to investigate whether exosomal EphA2 confers the invasive phenotype transfer from drug-resistant cells to drug-sensitive cells. As shown in Fig. 3a, EphA2 expression was downregulated in two drug-resistant cells expressing EphA2 shRNAs compared with the control shRNA. The knockdown of EphA2 did not affect the normal exosome secretion, whereas the expression of EphA2 in exosomes derived from EphA2 knockdown cells was reduced (Supplementary Fig. 5a, b). Moreover, transwell assay showed that exosomes from EphA2-silenced cells disable the migratory and invasive promoting effect in breast cancer cells (Fig. 3b, c). Consistently, the CM from EphA2-silenced drug-resistant cells failed to increase the motility of breast cancer cells (Supplementary Fig. 6a, b). To further determine the pro-invasive effect of exsomal EphA2, EphA2-overexpressed HEK-293T cells were established, and exosomes were collected (Fig. 3d). Consequently, exosomes from EphA2-expressing HEK-293T cells significantly promote the breast cancer cell migration and invasion ability compared with the control exosomes (Fig. 3e, f). Collectively, these findings indicate that exosomal EphA2 plays a critical role in transferring the invasive phenotype from drug-resistant cells to drug-sensitive cells.

### Exosomal EphA2 promotes migration and invasion of breast cancer cells by inducing Ephrin reverse signaling

**Fig. 4:**
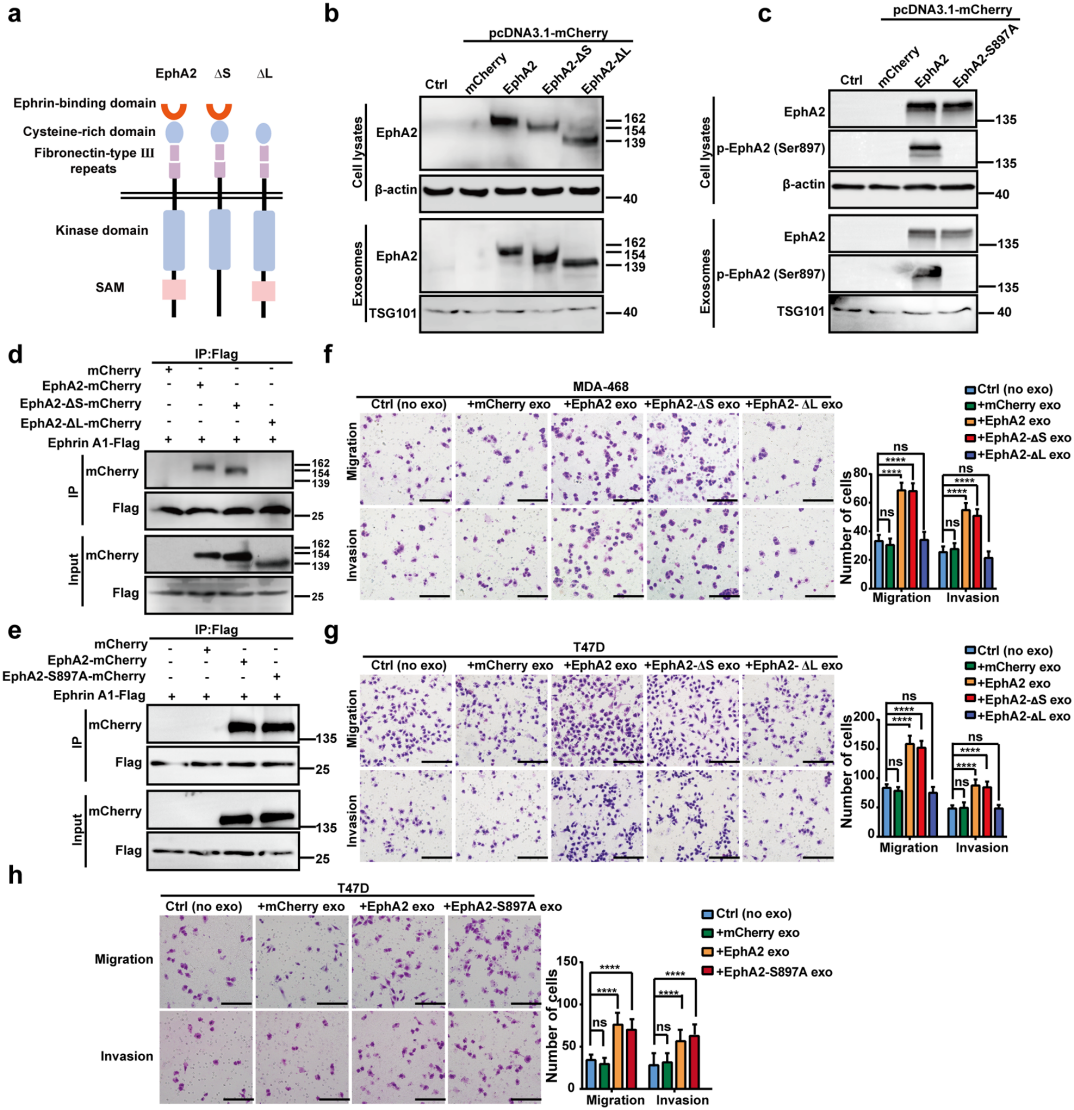
Exosomal EphA2 promotes migration and invasion of breast cancer cells through the reverse signaling pathway. **a** Schematic diagram of the structure of EpA2 mutants. The full-length EphA2 and its three mutants, EphA2-ΔL (deletion of the EphA2 ligand-binding domain), EphA2-ΔS (deletion of the EphA2 SAM domain), and EphA2-S897A (Ser897 mutated to alanine) were cloned into the pCDNA3.1-mCherry vector. **b** The expression of EphA2 and its mutants was detected in cell lysates and exosomes as measured by Western blotting assay. **c** The expression of EphA2 and EphA2-S897A was detected in cell lysates and exosomes as measured by Western blotting assay. β-actin was used as the loading control. **d**, **e** Flag-tagged Ephrin A1 was co-precipitated with mCherry-tagged EphA2, EphA2-ΔS, and EphA2-S897A but not with EphA2-ΔL mutants. **f, g** Exosomes carrying EphA2 and EphA2-ΔS, not EphA2-ΔL, promoted the migration and invasion abilities of breast cancer cells. All experiments were repeated at least three times. ****P < 0.0001 and ^ns^*P* > 0.05 indicate no statistical significance. **h** Exosomes carrying EphA2-S897A promoted the migration and invasion abilities of breast cancer cells. All experiments were repeated at least three times. ****P < 0.0001 and ^ns^*P* > 0.05 indicate no statistical significance. Scale bars: 200 μm.

**Fig. 5:**
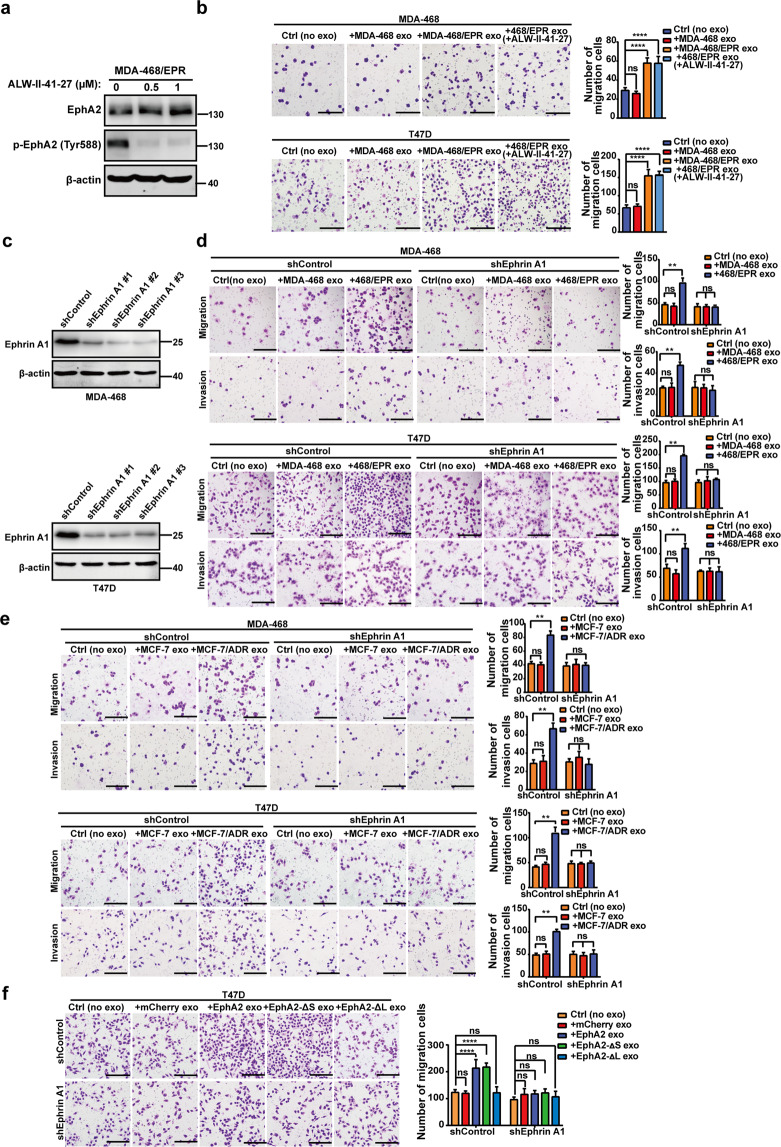
Drug-resistant cell-derived exosomal EphA2 promoted the migration and invasion of breast cancer cells by inducing Ephrin A1 reverse signaling. **a** Treatment with ALW-II-41-27 suppressed the phosphorylation of EphA2 at the Tyr588 site in drug-resistant breast cancer cells. **b** Exosomes derived from ALW-II-41-27-treated drug-resistant cells (DR-Exos) exerted migratory promoting effects on sensitive breast cancer cells. The drug-resistant cells were treated with ALW-II-41-27 (500 nM) for 12 h, and then the exosomes were collected and used for further assay. All experiments were repeated at least three times. ****P < 0.0001 and ^ns^*P* > 0.05 indicate no statistical significance. **c** Western blotting analysis of the expression of Ephrin A1 in MDA-MB-468 and T47D cells infected with lentivirus expressing control and Ephrin A1-specific shRNAs. **d**, **e** Exosomes derived from drug-resistant cells failed to promote the migration and invasion abilities in Ephrin A1 knockdown cells. All experiments were repeated at least three times. **P < 0.01 and ^ns^*P* > 0.05 indicate no statistical significance. **f** Exosomes carrying EphA2 and EphA2-ΔS failed to promote the migration of Ephrin A1 knockdown cells. All experiments were repeated at least three times. ****P < 0.0001 and ^ns^*P* > 0.05 indicate no statistical significance. Scale bars: 200 μm.

Unlike traditional receptor tyrosine kinases, the binding of EphA2 to its ligand Ephrin A1 can produce bidirectional signals. To investigate the mechanistic details through which exosomal EphA2 promoted the invasiveness of breast cancer cells, full-length EphA2 and its mutants, EphA2-ΔL, EphA2-ΔS, and EphA2-S897A, were constructed into pCDNA3.1-mCherry and transfected into HEK-293T cells. As shown in Fig. 4a–c, the expression of EphA2 and its mutants could be detected in cell lysates and exosomes. Next, Flag-tagged Ephrin A1 plasmid was constructed and then co-transfected with the EphA2 or mutant expression vectors into HEK-293T cells to investigate the interaction between EphA2 and Ephrin A1. As shown in Fig. 4d, e, Ephrin A1 was co-precipitated with EphA2, EphA2-ΔS, and EphA2-S897A but not with EphA2-ΔL mutants, indicating that EphA2 and its mutants were functioning as we expected. In addition, exosomes carrying EphA2-ΔS and EphA2-S897A could promote the migration and invasion of breast cancer cells, which were similar to exosomes carrying EphA2. However, exosomes carrying EphA2-ΔL failed to promote the migration and invasion of breast cancer cells (Fig. 4f–h). Therefore, these results indicated that the LBD domain was required for exosomal EphA2 to promote cell invasiveness. These data also indicated that exosomal EphA2 promoted the aggressive behavior of breast cancer cells through the reverse signaling pathway. To test this possibility, ALW-II-41-27, a small-molecule inhibitor of EphA2, was used to treat drug-resistant cells and then exosomes were treated on breast cancer cells. As shown in Fig. 5a, b, DR-Exos treated with ALW-II-41-27 still exerted profound migratory promoting ability, which indicated that exosomal EphA2 promoted breast cancer migration through EphA2-Ephrin A1 reverse signaling instead of the kinase-related forward signaling. To further confirm this hypothesis, we silenced Ephrin A1 expression in T47D and MDA-MB-468 cells (Fig. 5c). As expected, DR-Exos failed to promote the migration and invasion abilities in Ephrin A1-KD cells (Fig. 5d, e). We also treated Ephrin A1-KD cells with exosomes carrying EphA2 or its mutants. Transwell assay showed that these exosomes cannot promote the migration of Ephrin A1-KD cells (Fig. 5f). Collectively, these results indicated that exosomal EphA2 promoted breast cancer cell migration and invasion by inducing Ephrin A1 reverse signaling.

### Exosomal EphA2 derived from drug-resistant cells promotes breast cancer progression through ERK signaling

**Fig. 6:**
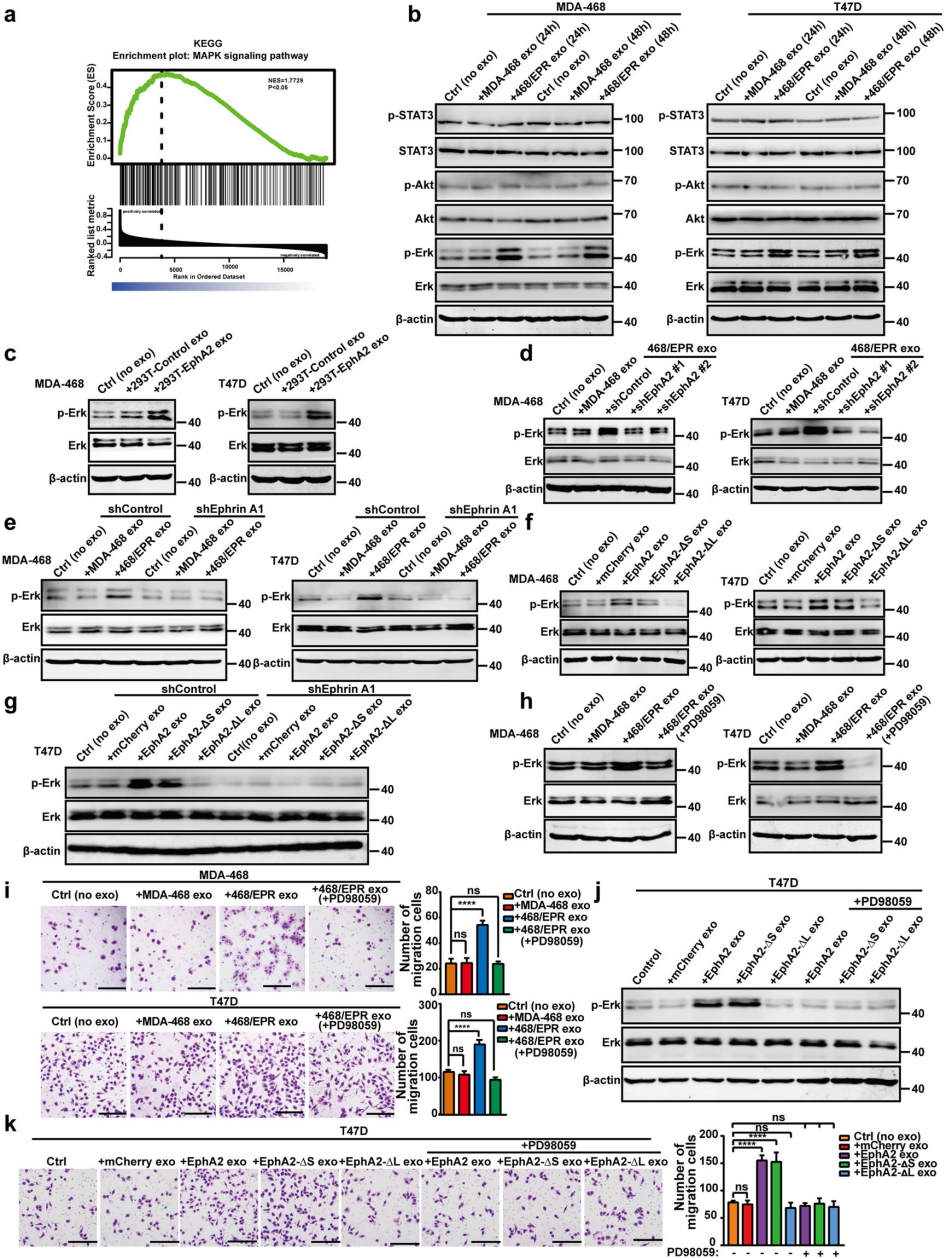
Drug-resistant cell-derived exosomal EphA2 promotes breast cancer progression through ERK signaling. **a** Gene set enrichment analysis (GSEA) showed that the expression of EphA2 significantly correlated with the MAPK signaling pathway based on the TCGA database. **b** Western blotting analysis of the expression total and phosphorylated Erk1/2, total and phosphorylated Akt, and total and phosphorylated STAT3 in two breast cancer cells treated with exosomes for 24 and 48 h. **c** Western blotting analysis of the expression of total and phosphorylated Erk1/2 in two breast cancer cells treated with EphA2-rich exosomes derived from HEK-293T cells for 24 h. β-actin was used as the loading control. **d** Exosomes from EphA2-stable silenced drug-resistant cells failed to induce an elevation of ERK1/2 phosphorylation. **e** DR-Exos failed to induce an upregulation of phosphorylated ERK1/2 in Ephrin A1 knockdown cells compared with control cells. **f** Exosomal EphA2 and its mutants EphA2-ΔS could induce upregulation of phosphorylated ERK1/2, whereas exosomal EphA2-ΔL failed to induce ERK1/2 phosphorylation. **g** Exosomal EphA2 or its mutants cannot induce an increase in ERK1/2 phosphorylation in Ephrin A1 knockdown cells. **h** PD98059 eliminated the phosphorylation of ERK after incubation with exosomes. **i** Inhibition of ERK signaling by PD98059 decreased the migration ability of breast cancer cells treated with DR-Exos. All experiments were repeated at least three times. ****P < 0.0001 and ^ns^*P* > 0.05 indicate no statistical significance. **j** Exosomes carrying EphA2 and EphA2-ΔS failed to induce an upregulation of phosphorylated ERK1/2 in the presence of PD98095 compared with control cells. **k** PD98059 blocked the migratory promoting effect of exosomes carrying EphA2 and EphA2-ΔS. All experiments were repeated at least three times. ****P < 0.0001 and ^ns^*P* > 0.05 indicate no statistical significance. Scale bars: 200 μm.

Gene set enrichment analysis (GSEA) indicated that EphA2 expression was positively correlated with the MAPK signaling pathway, which was closely related to tumor metastasis (Fig. 6a). As shown in Figs. 6b and S7a, the expression level of phosphorylated ERK1/2 (p-ERK1/2) in breast cancer cells was remarkably increased in the DR-Exos-treated group compared with that of the control and DS-Exos-treated group, whereas the phosphorylation of Akt and STAT3 was not changed. Moreover, EphA2-rich exosomes from HEK-293T cells induced an apparent increase in p-ERK1/2 in breast cancer cells (Fig. 6c). Furthermore, exosomes from EphA2-stable silenced drug-resistant cells failed to induce an increase of ERK1/2 phosphorylation (Figs. 6d and S7b). The phosphorylation of ERK1/2 is downstream of the EphA2-Ephrin A1 reverse signaling. Therefore, these findings indicated that exosomal EphA2-mediated reverse signaling promoted breast cancer progression. In confirming this hypothesis, the exosomes were used to treat Ephrin A1 knockdown cells. Consequently, DR-Exos failed to induce an upregulation of p-ERK1/2 in Ephrin A1-KD cells compared with control cells (Figs. 6e and S7c). Next, we determined the effect of exosomes carrying EphA2 and its mutants on the phosphorylation of ERK1/2 in breast cancer cells. As shown in Figs. 6f and S7d, exosomal EphA2 and its mutants EphA2-ΔS and EphA2-S897A could induce a profound ERK1/2 phosphorylation. On the contrary, exosomal EphA2-ΔL failed to promote ERK1/2 phosphorylation. These data indicated that exosomal EphA2 promoted ERK1/2 phosphorylation in a ligand-dependent manner. Consistently, exosomal EphA2 or its mutants cannot induce an increase in ERK1/2 phosphorylation in Ephrin A1 knockdown cells (Fig. 6g). Collectively, these findings indicated that exosomal EphA2 derived from drug-resistant cells promoted breast cancer progression through ERK signaling. To further confirm the above-mentioned findings, we pretreated breast cancer cells with the ERK inhibitor PD98059 and then added DR-Exos or DS-Exos. Western blotting assay showed that PD98059 eliminated the phosphorylation of ERK (Figs. 6h and S7e). Moreover, the inhibition of ERK signaling by PD98059 decreased the migration ability of breast cancer cells treated with DR-Exos (Figs. 6i and S7f). In addition, exosomes carrying EphA2 and EphA2-ΔS failed to induce an upregulation of p-ERK1/2 in the presence of PD98095 compared with control cells (Fig. 6j). Consequently, PD98059 blocked the pro-migratory effect of exosomes carrying EphA2 and EphA2-ΔS (Fig. 6k). These results indicated that exosomal EphA2 promoted the aggressive behavior of breast cancer cells by activating the ERK1/2 pathway, which was downstream of EphA2-Ephrin A1 reverse signaling.

### Exosomal EphA2 promoted breast cancer cell metastasis in vivo

**Fig. 7:**
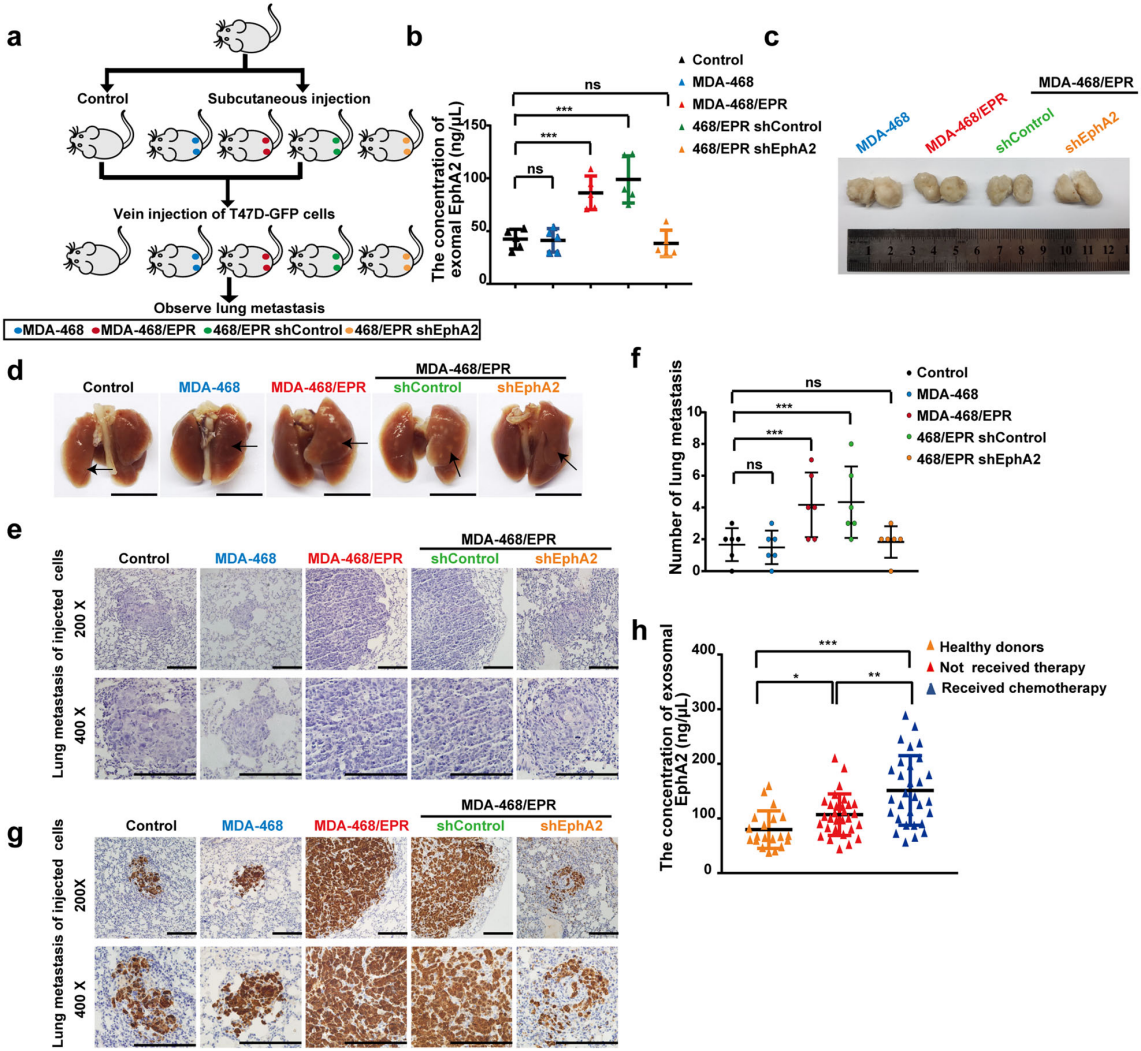
Exosomal EphA2 promotes breast cancer cell metastasis in vivo. **a** Schematic diagram of the in vivo experimental design. **b** Exosomal EphA2 protein was significantly upregulated in plasma from the MDA-MD-468/EPR and MDA-MD-468/EPR-sh control groups compared with the MDA-MD-468 and MDA-MD-468/EPR-EphA2-KD groups. ***P < 0.001 and ^ns^*P* > 0.05 indicate no statistical significance. **c** Representative images of subcutaneous tumor formed in mice. **d** The drug-resistant cell inoculated group showed more metastatic foci on the mice lung surface than that in the drug-sensitive cell inoculated group. Scale bars: 1 cm. **e, f** H & E staining showed that the number of tumor metastatic foci in the lung tissue was significantly higher in the drug-resistant cell inoculated group than in the drug-sensitive cell inoculated group. Data are shown as mean ± SD. Statistical analysis was performed by one-way ANOVA. ***P < 0.001 and ^ns^*P* > 0.05 indicate no statistical significance. Scale bars: 200 μm. **g** The metastatic foci in the lungs of mice were investigated using immunohistochemical (IHC) staining with anti-GFP antibodies. Scale bars: 200 μm. **h** ELISA assays showed that the concentration of exosomal EphA2 in plasma was collected from healthy donors (*n* = 20), early-stage breast cancer patients without any systemic treatment (*n* = 30), and advanced patients received at least one prior line of systemic therapy (*n* = 30). Data are shown as mean ± SD. Statistical analysis was performed by one-way ANOVA. *P < 0.05, **P < 0.01, ***P < 0.001, and ^ns^*P* > 0.05 indicate no statistical significance.

We first established xenograft tumor models by subcutaneous injection of MDA-MB-468 cells, MDA-MB-468/EPR cells, control, and EphA2-stable knockdown cells into the fat pad of SCID mice to investigate the function of exosomal EphA2 on the metastatic potential of breast cancer cells in vivo. Four weeks after inoculations, the volume of the tumors reached ~1 cm^3^, and the tumors in all groups were similar in size. Next, we injected EGFP-labeled T47D cells into the xenograft tumor models via the tail vein (Fig. 7a). Two months after injection, the mice were anesthetized, and their peripheral blood was collected. Then, the plasma was separated and used to determine the content of exosomal EphA2 protein. ELISA showed that exosomal EphA2 protein was significantly upregulated in the plasma from the MDA-MD-468/EPR and MDA-MD-468/EPR-EphA2-sh control groups compared with the MDA-MD-468 and MDA-MD-468/EPR-EphA2-KD groups (Fig. 7b). This result indicated that drug-resistant tumor cells could release exosomal EphA2 into the peripheral circulation. Next, the mice were sacrificed, and the tumor was isolated. As shown in Fig. 7c, the tumor size in all groups showed comparable size. An apparent decrease of metastatic foci was observed in the lung surface of the EphA2-silenced group compared with the control group (Fig. 7d). H & E staining showed that the number of tumor metastatic foci in the lung surface was significantly higher in the drug-resistant cell inoculated group than in the drug-sensitive cell inoculated group (Fig. 7e, f). In addition, immunohistochemistry (IHC) using anti-GFP antibody confirmed that all the metastatic foci in the lungs were GFP-positive, indicating that those foci were formed by the T47D-GFP cells but not the pre-subcutaneously injected tumor cells (Fig. 7g). Collectively, these results suggested that exosomal EphA2 could promote breast cancer cell metastasis in vivo.

### Plasma exosomal EphA2 is an indicator of drug resistance and metastasis

To determine whether the elevated level of exosomal EphA2 in plasma correlated with the prognosis of cancer patients, we collected circulating exosomes in the plasma of healthy donors and breast cancer patients (with or without chemotherapy), and then the level of the exosomal EphA2 was investigated using the ELISA method. The level of circulating exosomal EphA2 in the plasma of breast cancer patients was significantly higher than that of healthy donors (Fig. 7h). Moreover, the EphA2 levels in the plasma of breast cancer patients receiving chemotherapy were significantly higher than that at the time of initial diagnosis. Thus, our clinical data showed that a high level of EphA2 in circulating exosomes was associated with cancer progression.

## Discussion

The primary findings of this study support a model, in which exosomes derived from drug-resistant cells mediate cancer cell–cell communications and promote the invasion and metastasis of sensitive breast cancer cells. EphA2 is enriched in exosomes derived from drug-resistant cells and confers the invasive phenotype transfer from drug-resistant cells to sensitive cells. Exosomal EphA2 activates ERK1/2 signaling through the ligand Ephrin A1-dependent reverse pathway rather than the forward pathway, thereby promoting breast cancer progression. Collectively, these results highlight the key functional role of exosomal EphA2 in the transmission of aggressive phenotype between cancer cells that do not rely on direct cell–cell contact. Our study also suggests that the increase of EphA2 in DR-Exos may be an important mechanism of chemotherapy/drug resistance-induced breast cancer progression.

Exosome-mediated cancer cell–cell communications in tumor microenvironment play a key role in promoting cancer progression^46–49^. Tumor cells release exosomes to educate stromal cells, thereby inducing niche formation before distant metastasis. Exosomes transfer messages from stromal cells to cancer cells and contribute to tumor growth, dissemination, and therapy resistance^28,29,50–54^. PC cells can produce exosomes to act on neighboring tumor cells and promote chemo-resistance^32,33^. In this study, we have observed that exosomes of resistant breast cancer cells not only enhance resistance to chemotherapeutic drugs of sensitive cells, but also promote the invasive and metastatic behavior of breast cancer cells. These results indicate that apart from the interaction between cancer and stromal cells, communications among cancer cells also promote tumor progression. A recent study has demonstrated that chemotherapy can promote the release of exosomes from tumor cells, thereby facilitating the metastasis of cancer cells. In addition, drug-resistant cancer cells always exhibit highly aggressive phenotypes^19–21,55,56^. Collectively, these findings indicate that certain tumor cells can not only acquire stronger adaptability through evolution after being subjected to external stress (such as anti-tumor drugs), but also promote the survival and invasion of other cancer cells through cell-to-cell communication.

One of our findings is that exosomal EphA2 confers the invasive phenotype transfer from drug-resistant cells to drug-sensitive cancer cells. The well-known function of EphA2 is to interact with cell surface-anchored ligand Ephrin A1 upon cell–cell contact, and forms a pivotal cell–cell communication system^34,35,38–40,57^. Unlike previous reports, our data show a novel EphA2-Ephrin A1 signal transmission system occurring at the exosome–cell surface that does not involve direct cell–cell contact. Interestingly, EphA2 is highly expressed in DR-Exos, but not in DS-Exos and drug-sensitive cells, whereas the level of Ephrin A1 is higher in drug-sensitive cells than in drug-resistant cells (Supplementary Fig. 8). Thus, exosomal EphA2 functions as paracrine signaling in cell–cell communications. The presence of EphA2 in exosomes enables the EphA2/Ephrin A1 system to travel to distant locations and perform long-range intercellular communication. To date, little information is available regarding the functional significance of the exosomal EphA2. In this study, exosomes rich in EphA2 significantly promote the invasion of breast cancer cells, whereas exosomes without EphA2 fail to enhance the invasive behavior in breast cancer cells. Hence, our results indicate that the exosomal transmission of EphA2 from drug-resistant cells to drug-sensitive cells plays an important role to promote the progression of breast cancer.

The mechanism whereby exosomal EphA2 enhances the aggressive behavior of breast cancer cells needs further investigation. Theoretically, exosomal EphA2 can act on recipient cells in a ligand-dependent or independent manner through the forward or reverse signaling pathways^35^. Herein, exosomes carrying EphA2 or its mutants can promote the invasive potential of sensitive breast cancer cells, whereas exosomes carrying EphA2-ΔL lost the ability to promote cell invasion. These results indicate that EphA2 binding to Ephrin A1 is necessary for the pro-invasive effect of exosomal EphA2. In addition, inhibiting EphA2 kinase activity by using inhibitors shows profound invasion promotion ability, indicating that the pro-invasive effect of exosomal EphA2 is not through the kinase-dependent forward signaling pathway. Moreover, exosomes carrying EphA2-S897A also show a significant invasive promoting effect on breast cancer cells, indicating that the kinase-independent forward signaling is not involved in this effect. Therefore, these data suggest a possibility that exosomal EphA2 promotes the invasion of sensitive cells through EphA2-Ephrin A1 reverse signaling. Consistent with this hypothesis, exosomes carrying EphA2 or its mutants fail to promote the invasive ability of Ephrin A1-silenced cells. Hence, the binding of exosomal EphA2 to Ephrin A1-induced reverse signaling promotes aggressive behavior in breast cancer.

The detailed mechanisms downstream of exosomal EphA2-Ephrin A1 reverse signaling remain to be settled. In this study, DR-Exos or EphA2-expressing HEK-293T cells can induce an increase of p-Erk1/2 in sensitive cells, whereas exosomes from EphA2-silenced cells fail to induce the elevation of ERK1/2 phosphorylation, indicating a possible involvement of ERK1/2 downstream of EphA2-Ephrin A1 reverse signaling. In addition, exosomal EphA2 fails to induce an upregulation of p-ERK1/2 in Ephrin A1-silenced cells, and exosomal EphA2-ΔL also fails to promote ERK1/2 phosphorylation in sensitive cells. Thus, our data show that exosomal EphA2 promotes ERK1/2 phosphorylation in an Ephrin A1-dependent manner. Consistently, a recent study has shown that exosomes from senescent cells can activate ERK1/2 through EphA2-Ephrin A1 reverse signaling^43^. Moreover, GSEA indicates that EphA2 expression is positively correlated with the MAPK/ERK signaling pathway. Collectively, our results suggest that exosomal EphA2 derived from drug-resistant cells promotes breast cancer progression through the ERK pathway downstream of EphA2-Ephrin A1 reverse signaling.

In summary, our results indicated the key function of exosomal EphA2 involved in the crosstalk between drug resistance and cancer progression. Drug-resistant cells can promote the invasion and metastasis of sensitive cells by transferring exosomal EphA2, thereby activating the Ephrin A1-dependent reverse pathway rather than the forward pathway independent of direct cell–cell contact. Thus, the exosomal EphA2-mediated intercellular communications between drug-resistant cells and sensitive cells may be an important mechanism of drug resistance-induced breast cancer progression. Nevertheless, this study cannot exclude that other molecules enriched in exosomes also play a role in promoting cancer aggressiveness. One of the notable molecules is ALPP (Alkaline Phosphatase, Placental), which is highly enriched in DR-Exos than in DS-Exos. A high ALPP level has been observed in many types of cancers^58,59^. However, its possible role in cancer progression has not yet been determined. In future studies, whether exosomal ALPP is necessary for resistance-induced breast cancer metastasis needs to be further explored.

## Materials and methods

### Patient and specimen collection

Plasma samples from 60 patients with breast cancer (female) and 20 healthy volunteers (female) were collected at Tianjin Medical University Cancer Institute and Hospital between September 2019 and November 2019. Thirty cases of plasma were collected from patients with early-stage operable invasive breast cancer, which did not receive any systemic treatment before specimen collection. Another 30 cases of plasma were collected from patients with advanced breast cancer; these patients have received at least one prior line of systemic therapy. The plasma samples were also collected from 20 healthy volunteers with matching ages and genders to the patients. Table 1 provides the clinicopathological characteristics of these patients enrolled in this study. The plasma samples were centrifuged at 1000 × *g* for 10 min. Plasma specimens were stored at −80 °C. This study was approved by the Ethics Committee of Tianjin Medical University Cancer Institute and Hospital and was conducted in accordance with the Declaration of Helsinki.

**Table 1:** Clinicopathological characteristics of breast cancer patients enrolled in this study.

Characteristics	Healthy donors (*n* = 20)	Therapy	
		Not received (*n* = 30)	Received (*n* = 30)
Median age at diagnosis (years)		53 (31–83)	56 (30–80)
Sex			
Male	0	0	0
Female	20	30	30
Chemotherapy			
Received		0	30
Not Received		30	0
Prior lines of treatment			
1 line		0	1
2 line		0	6
3 + line		0	23

### Cell lines and cell culture

Human embryonic kidney 293T (HEK-293T) and human breast cancer cell lines MDA-MB-468 and T47D were obtained from American Type Culture Collection. The Epirubicin-resistant cell line MDA-MB-468/EPR was established by our group in a previous study^20,60^. Human breast cancer cell line MCF-7 and its adriamycin-resistant cell line MCF-7/ADR was provided by Henry Ford Hospital in Detroit, Mississippi, USA. T47D, MCF-7, and MCF-7/ADR cells were cultured in RPMI-1640 medium (Hyclone, Logan, UT, USA). MDA-MB-468 and MDA-MB-468/EPR cells were cultured in DMEM/F12 medium (Hyclone, Logan, UT, USA). HEK-293T cells were cultured in DMEM/high-glucose medium (Hyclone, Logan, UT, USA). All media were supplemented with 10% fetal bovine serum (FBS, Gibco, Carlsbad, CA, USA). FBS exosomes were depleted by ultracentrifugation at 100,000 × *g* for 16 h, followed by sterile filtering with 0.22 µm filters, to exclude the influence of serum exosomes on the cell functional activities. The CM was prepared by incubating cells for 12 h in serum-free medium and filtered through a 0.22 µm filter to remove cells and cellular debris.

### Exosomes isolation from cells

Exosomes were obtained from cell culture medium as previously described^61^. In brief, the cell culture medium was collected and centrifuged at 300 × *g* for 10 min to remove cells, and then the supernatant was centrifuged at 3000 × *g* for 10 min to remove cell debris, followed by centrifugation at 10,000 × *g* for 30 min at 4 °C to remove large vesicles. The supernatant was further centrifuged at 100,000 × *g* for 90 min at 4 °C. The exosomal pellets were resuspended in PBS and then centrifuged again at the same speed. The purified exosomes were further characterized and analyzed.

### Exosomes isolation from human plasma samples

In the method established by Kahlert et al.^62,63^, 500 μL of plasma samples was thawed on ice. The plasma was diluted in 12.5 mL PBS and then ultracentrifuged at 160,000 × *g* overnight at 4 °C. Next, the exosomal pellets were washed in PBS, followed by a second step of ultracentrifugation at 160,000 × *g* at 4 °C for 2 h. The supernatant was discarded, and the exosomal pellets were resuspended in 100 μL of PBS.

### Characterization of purified exosomes

For transmission electron microscopy (TEM) analysis, exosomes suspended in PBS were dropped on formvar carbon-coated grid, incubated for 5 min, and then stained with 2% phosphotungstic acid for 2 min. The grid was dried in air for 5–10 min. Images were obtained using a TEM device (HT7700, HITACHI Company) at 80 kV. In addition, the size and concentration of exosomes were tracked using the NanoSight NS300 device (Malvern Instruments).

### PKH26 staining

Exosomes were stained with the PKH26 Red Fluorescent Cell Linker Kit (Sigma-Aldrich, MO, USA), according to the manufacturer’s instructions with minor modifications. First, exosomes were diluted in 250 μL of diluent C. Second, 1 μL of PKH26 dye was added to another 250 μL of diluent C, and then the exosomes and dye were mixed together by gently pipetting, followed by incubating at room temperature for 3 min. Then, 500 μL of FBS was added to the mixture to quench the excess dye. Finally, the sample was diluted in 12.5 mL PBS and ultracentrifuged at 100,000 × *g* at 4 °C for 90 min, followed by resuspending in a fresh medium.

### Enzyme-linked immunosorbent assays (ELISA)

96-well ELISA plates (Biolegend, CA, USA) were coated with 50 µL/well of a 1:100 dilution of anti-human CD81 antibodies (R&D Systems, MN, USA) and incubated overnight at 4 °C. After washing three times with PBS, the plates were blocked with 5% BSA in PBS with 0.05% Tween-20 (PBST) at room temperature for 2 h (50 µL/well). Then, plasma exosome samples (100 µL/well) were added to the plate and incubated overnight at 4 °C. After three washes with PBST, 50 µL of anti-human EphA2 antibodies (0.2 μg/mL, Novus, MN, USA) was added and incubated at 37 °C for 1 h. The plates were then washed three times with PBST and incubated with horseradish peroxidase (HRP)-conjugated secondary antibody (BIORAD, CA, USA) at room temperature for 1 h (100 μL/well). After three times final washes with PBST, plates were incubated with 50 μL/well TMB reagent (Cell Signaling Technology, MA, USA) at room temperature for 10–15 min, followed by the addition of 50 μL/well of stop solution (2 M H_2_SO_4_). The absorbance was read at 450 nm using a micro-ELISA reader.

### Western blotting

Western blotting was performed as described previously^22^. In brief, whole-cell lysates or exosomal proteins were separated by SDS–PAGE and transferred onto PVDF membranes. The membranes were blocked with 5% milk for 1 h at room temperature and then incubated with the corresponding primary antibodies overnight at 4 °C. The following antibodies were used: TSG101 (sc-136111, Santa Cruz, CA, USA), CD81 (sc-23962, Santa Cruz), Alix (92880, CST, MA, USA), ERK (4695, CST), p-ERK (4370, CST), Akt (9272, CST), p-Akt (4051, CST), STAT3 (12640, CST), p-STAT3 (9145, CST), EphA2 (6997, CST), EphA2 (398832, Santa Cruz), Rab27a (ab55667, Abcam, MA, USA), and β-actin (A1978, Sigma-Aldrich, MO, USA). After washing three times with TBST, the membrane was incubated with HRP-conjugated secondary antibodies at room temperature for 1 h. The signals were visualized with the ECL kit. CD81, Alix, and TSG101 were used as exosomal markers. β-actin was used as a loading control.

### Wound healing and transwell assay

Wound healing assay was performed as described previously^22^. Cells were cultured to confluence in 6-well plates and then treated with CM for 12 h. Then, a 10 μL pipette tip was used to scrape a wound on the cell monolayer. After washing two times with PBS to remove the detached cells, the medium was replaced with fresh CM containing 2% exosome-depleted FBS. The plates were then incubated at 37 °C for 48 h in 5% CO_2_. The width of the wound gap was captured under an inverted microscope. Transwell assay was performed by using a Boyden chamber with a pore size of 8 μm as described previously^22^. The cells were pre-treated with exosomes for 24 h to study the effect of exosomes on migration and invasion of cancer cells. Then, transwell assays were performed with or without Matrigel. For cell migration assay, 5 × 10^4^ cells suspended in 200 μL of serum-free medium were loaded onto the upper chambers. 600 μL of medium with 10% FBS was added into the lower chamber. For cell invasion assay, 1 × 10^5^ cells suspended in 200 μL of serum-free medium were loaded onto the upper chambers coated with Matrigel. After incubation at 37 °C for 24 h, the migrated or invaded cells were fixed, stained, and captured by a microscope at ×200.

### Immunofluorescence assay

Immunofluorescence assay was carried out as described previously^20^. In brief, cells were seeded in 12-well plates containing glass coverslips and incubated at 37 °C for 12 h in 5% CO_2_. Afterward, the cells were fixed with 4% PFA/PBS and permeabilized with 0.1% Triton X-100 in PBS for 10 min, followed by blocking with 3% BSA/PBS for 1 h. Then, the cells were incubated with primary antibodies overnight at 4 °C. After washing three times with PBS, the cells were then stained with Alexa Fluor 488-conjugated secondary antibodies at room temperature for 1 h in the dark, followed by nuclear staining by using 1 ng/mL of DAPI. The coverslips were mounted and observed by using a laser scanning confocal microscope (Zeiss Axio Imager).

### Vector construction and stable transfection

EphA2, Ephrin A1, and Rab27a-specific shRNA sequences were subcloned into a lentiviral vector, pLko.1-hygromycin, in the BamH Ӏ and Age Ӏ cloning sites. The sequences of the shRNAs are listed in Supplementary Table 1. The EphA2-coding sequences were cloned from cDNA plasmid purchased from ORIGENE (Beijing, China) using polymerase chain reaction (PCR). The truncation mutants tagged with mCherry (EphA2-ΔS and EphA2-ΔL) were created by overlapping PCR and cloned into a linearized pCDNA3.1 vector using a ClonExpress II one-step cloning kit (Vazyme Biotech, Nanjing, China). The point mutation of mCherry-tagged EphA2 (S987A) was introduced by PCR-based site-directed mutagenesis and cloned into the pCDNA3.1 vector. The Flag-tagged Ephrin A1 was amplified from human cDNA using PCR and cloned into a linearized pCDNA3.1 vector using a ClonExpress II one-step cloning kit (Vazyme Biotech). All the plasmids were confirmed by restriction digestion and DNA sequencing. The primers used for amplification of Ephrin A1, EphA2, and its mutants are listed in Supplementary Table 2. Plasmid transfections were performed using Lipofectamine 3000 (Thermo Fisher Scientific, CA, USA), according to the manufacturer’s instructions.

### Co-immunoprecipitation assay

Co-immunoprecipitation assay was performed as described previously^60^. In brief, cells were washed three times with ice-cold PBS, solubilized with lysis buffer (40 mM Tris, 150 mM NaCl, 1% Triton X-100, 50 mM NaF, 5 mM Na_3_VO_4_, 2 mM EDTA, and protease inhibitor cocktail), and incubated on ice for 1 h. Lysates were then centrifuged at 12,000 × *g* for 15 min at 4 °C. The supernatants were pre-cleared for 1 h with protein A-conjugated agarose beads, followed by incubation with 1 μg of anti-Flag antibody overnight at 4 °C. The immunocomplex was incubated with protein A agarose beads for 1 h at room temperature. The beads were then washed three times with cell lysis buffer. The final pellets were resuspended with 2× SDS sample buffer. The samples were then analyzed by Western blotting.

### Mass spectrometric analysis and bioinformatics analysis

The exosome samples were prepared in three biological replicates from the CM of MDA-MB-468 and MDA-MB-468/EPR cells. Then, the exosome samples were processed for tandem mass tag (TMT) quantitative proteomic analysis by PTM BioLab (Hangzhou, China). The detailed procedure was as following: Exosome samples were sonicated in lysis buffer (8 M urea, 1% Protease Inhibitor Cocktail) on ice. The protein solution was reduced with 5 mM DTT at 56 °C for 30 min, and alkylated with 11 mM iodoacetamide at room temperature in dark for 15 min. Afterward, the samples were incubated with trypsin at a protein-to-trypsin mass ratio of 50:1 and 100:1 for the first digestion overnight and a second 4 h digestion, respectively. The tryptic peptides were reconstituted in 0.5 M TEAB and incubated with TMT reagent for 2 h at room temperature according to the manufacturer’s protocol for TMT kit. Then the peptides were fractionated into fractions by high pH reverse-phase HPLC using Thermo Betasil C18 column (5 μm particles, 10 mm ID, 250 mm length). Next, the tryptic peptides were dissolved in 0.1% formic acid (solvent A), directly loaded onto a home-made reversed-phase analytical column (15-cm length, 75 μm i.d.). The gradient was comprised of an increase from 6 to 23% solvent B (0.1% formic acid in 98% acetonitrile) over 26 min, 23 to 35% in 8 min and climbing to 80% in 3 min then holding at 80% for the last 3 min, all at a constant flow rate of 350 nL/min on an EASY-nLC 1000 UPLC system. The peptides were subjected to NSI source followed by tandem mass spectrometry (MS/MS) in Q ExactiveTM Plus (Thermo) coupled online to the UPLC. The electrospray voltage applied was 2.0 kV. The *m/z* scan range was 350–1550 for full scan, and intact peptides were detected in the Orbitrap at a resolution of 60,000. The scanning range of the secondary mass spectrum is fixed at 100 *m/z*, and the secondary scanning resolution is set to 15,000. The data acquisition mode uses the data-dependent scanning (DDA) program, that is, the first 20 peptide precursor ions with the highest signal intensity are selected to enter the HCD collision cell and fragmented using 32% of the fragmentation energy after the first scan. For grade mass spectrometry analysis, automatic gain control (AGC) was set at 5E4. Fixed first mass was set as 70 m/z. The resulting MS/MS data were processed using Maxquant search engine (v.1.5.2.8). Search parameter settings were as follows: the database is Human_SwissProt_1808 (20387 sequences), an anti-database is added to calculate the false positive rate (FDR) caused by random matching, and a common contamination library is added to the database to eliminate the contamination of the protein in the identification results influences. Trypsin/P was specified as a cleavage enzyme allowing up to two missing cleavages. The mass tolerance for precursor ions was set as 20 ppm in the First search and 5 ppm in Main search, and the mass tolerance for fragment ions was set as 0.02 Da. Carbamidomethyl on Cys was specified as fixed modification and acetylation modification and oxidation on Met were specified as variable modifications. For GSEA, Pearson’s correlation value was calculated between EPHA2 and all protein-coding genes in TCGA-BRCA RNAseq data and subjected to WebGsetalt database (http://www.webgestalt.org/). The GSEA was performed using the KEGG gene sets.

### In vivo metastasis assay

Four-week-old female SCID mice were purchased from Beijing Charles River (Beijing, China). All animal work procedures were approved by the Animal Ethical and Welfare Committee of Tianjin Medical University Cancer Institute and Hospital. The mice were randomly allocated to five groups (six mice/group). 5 × 10^6^ cells (MDA-MD-468, MDA-MD-468/EPR, control, and EphA2-stable knockdown MDA-MD-468/EPR cells) were subcutaneously injected into the mammary fat pad of SCID mice. After injection, mouse weight and tumor size were measured once a week, and the subcutaneous tumor volume was calculated via the standard modified formula volume (cm^3^) = 1/2 (height^2 ^× length). 1 × 10^6^ GFP-labeled T47D cells were injected into SCID mice via tail veins when the tumor size reached 1 cm^3^. Two months after injection, the mice were anesthetized, and their peripheral blood was collected. Then, the mice were sacrificed, and the lung tissues were dissected and fixed in 4% neutral-buffered formalin. Afterward, the tissues were paraffin-embedded for H&E staining and immunohistochemical staining. The metastatic nodules were counted by H&E-stained tissues. Immunohistochemical staining was performed with anti-GFP antibodies to confirm the origin of the metastatic cancer cells.

### Statistical analysis

All data were presented as mean ± SD of at least three independent experiments. GraphPad Prism 7.0 software was used to conduct statistical analysis. One-way or two-way ANOVA tests were performed for statistical analysis of the differences among groups. *P* < 0.05 was considered statistically significant.

## Supplementary information

supplementary Figure Legends

Supplementary tables

Supplementary Fig 1

Supplementary Fig 2

Supplementary Fig 3

Supplementary Fig 4

Supplementary Fig 5

Supplementary Fig 6

Supplementary Fig 7

Supplementary Fig 8
