# Supplementary material for: Drug-resistant cancer cell-derived exosomal EphA2 promotes breast cancer metastasis via the EphA2-Ephrin A1 reverse signaling
Source: Cell Death Dis. 2021 Apr 20;12(5):414. doi: 10.1038/s41419-021-03692-x (PMC8058342; doi:10.1038/s41419-021-03692-x)
Supplement: Supplementary file 1 — supplementary Figure Legends [file 41419_2021_3692_MOESM1_ESM.docx]

**Supplementary Figure legends**

**Supplementary Figure S1.** The stained exosomes can be endocytosed into the recipient cells. (a-b) Cells expressing GFP were incubated with PKH-26 labeled exosomes from drug-sensitive cells and drug-resistant cells. Scale bars: 200 μm.

**Supplementary Figure S2.** Drug-resistant cells can transfer chemo-resistance to T47D cells through transmitting P-glycoprotein (P-gp) via exosomes. (a) The expression of P-gp in T47D cells was significantly increased after incubating with exosomes derived from MCF-7/ADR. (b) T47D cells showed enhanced chemoresistance to EPI after treatment with exosomes derived from MCF-7/ADR cells. *****P* < 0.0001

**Supplementary** **Figure S3.** MS/MS spectra of several representative proteins.

**Supplementary** **Figure S4.** The expression of ALPP, ABCB1, ACE2, IVL, SERPINH1, ANXA1, ANPEP in the exosomes derived from drug-resistant cells was significantly higher than exosomes from the parental cells.

**Supplementary** **Figure S5.** Knockdown of EphA2 did not affect the secretion of exosomes. ^ns^*P* > 0.05 indicate no statistical significance.

**Supplementary** **Figure S6.** The CM from EphA2 silenced drug-resistant cells failed to increase the motility of T47D and MDA-MD-468 cells. *****P* < 0.0001. Scale bars: 200 μm.

**Supplementary Figure S7.** Drug-resistant cell-derived exosomal EphA2 promotes breast cancer progression through ERK signaling. (a) Western blotting analysis of the expression total and phosphorylated Erk1/2, total and phosphorylated Akt, and total and phosphorylated STAT3 in two breast cancer cells treated with exosomes for 24 and 48 h. (b) Exosomes from EphA2-stable silenced drug-resistant cells failed to induce an elevation of ERK1/2 phosphorylation. (c) DR-Exos failed to induce an upregulation of phosphorylated ERK1/2 in Ephrin A1 knockdown cells compared with control cells. (d) Exosomal EphA2 and its mutants EphA2-S897A could induce an upregulation of phosphorylated ERK1/2 in T47D cells. (e) PD98059 eliminated the phosphorylation of ERK after incubation with exosomes. (f) Inhibition of ERK signaling by PD98059 decreased the migration ability of breast cancer cells treated with DR-Exos. All experiments were repeated at least three times. *****P* < 0.0001 and ^ns^*P* > 0.05 indicate no statistical significance. Scale bars: 200 μm.

**Supplementary Figure S8.** The level of Ephrin A1 is higher in drug-sensitive cells than in drug-resistant cells.
