# Supplementary material for: Drug-resistant cancer cell-derived exosomal EphA2 promotes breast cancer metastasis via the EphA2-Ephrin A1 reverse signaling
Source: Cell Death Dis. 2021 Apr 20;12(5):414. doi: 10.1038/s41419-021-03692-x (PMC8058342; doi:10.1038/s41419-021-03692-x)
Supplement: Supplementary file 2 — Supplementary tables [file 41419_2021_3692_MOESM2_ESM.docx]

Supplementary Table 1 siRNA sequences used in this study

| Name | Sequence |
| --- | --- |
| shEphA2 #1 | 5′-CTATTCTGTCAGTGTTAAA-3′ |
| shEphA2 #2 | 5′-GATAAGTTTCTATTCTGTCAG-3′ |
| shRab27a #1 | 5′-GCTGCCAATGGGACAAACATA-3′ |
| shRab27a #2 | 5′-CAGGAGAGGTTTCGTAGCTA-3′ |
| shEphrin-A1 #1 | 5′-AGAGGTGCGG GTTCTACATAG-3′ |
| shEphrin-A1 #2 | 5′-GTCTTCTGGAACAGTTCAAAT-3′ |
| shEphrin-A1 #3 | 5′-ATGCCACACCTGGCCTTAAAG-3′ |

Supplementary Table 2 Primers used in this study

| Name | Primers |
| --- | --- |
| Ephrin-A1 | Upper: 5′-TAGTCCAGTGTGGTGGAATTCGCCACCATGGAGTTCCTCTGGGCCC-3′  Lower: 5′-GGTTTAAACGGGCCCTCTAGATCACTTATCGTCGTCATCCTTGTAATCC  GGGGTTTGCAGCAGCAG-3′ |
| EphA2 | Upper: 5′-GAGAAAGCTTGCCACCATGGAGCTCCAGGCAGCC-3′  Lower: 5′-GAGAACTAGTGATGGGGATCCCCACAG-3′ |
| EphA2-ΔS | Upper: 5′-TAGTCCAGTGTGGTGGAATTCGCCACCATGGAGCTCCAGG-3′  Lower: 5′- GAGAACTAGTGCTTCCTCCTCCTCCCCCTCCGAGCCGCTCGTG-3′ |
| EphA2-ΔL | Upper: 5′-GTCCAGTGTGGTGGAATTCGCCACCATGGGCCTGGCCCACTTCCCT-3′  Lower: 5′-GAGAACTAGTGCTTCCTCCTCCTCCGATGGGGATCCCCACAG-3′ |
| EphA2-S898A | Upper: 5′-TCCCCGCAACGAGCGGCTCGGAGGGGGTGCCCTTCCGCAC-3′  Lower: 5′- AGCCGCTCGTTGCGGGGAGCCGGATAGACACGC-3′ |
