# Supplementary figures and images for: Drug-resistant cancer cell-derived exosomal EphA2 promotes breast cancer metastasis via the EphA2-Ephrin A1 reverse signaling
Source: Cell Death Dis. 2021 Apr 20;12(5):414. doi: 10.1038/s41419-021-03692-x (PMC8058342; doi:10.1038/s41419-021-03692-x)

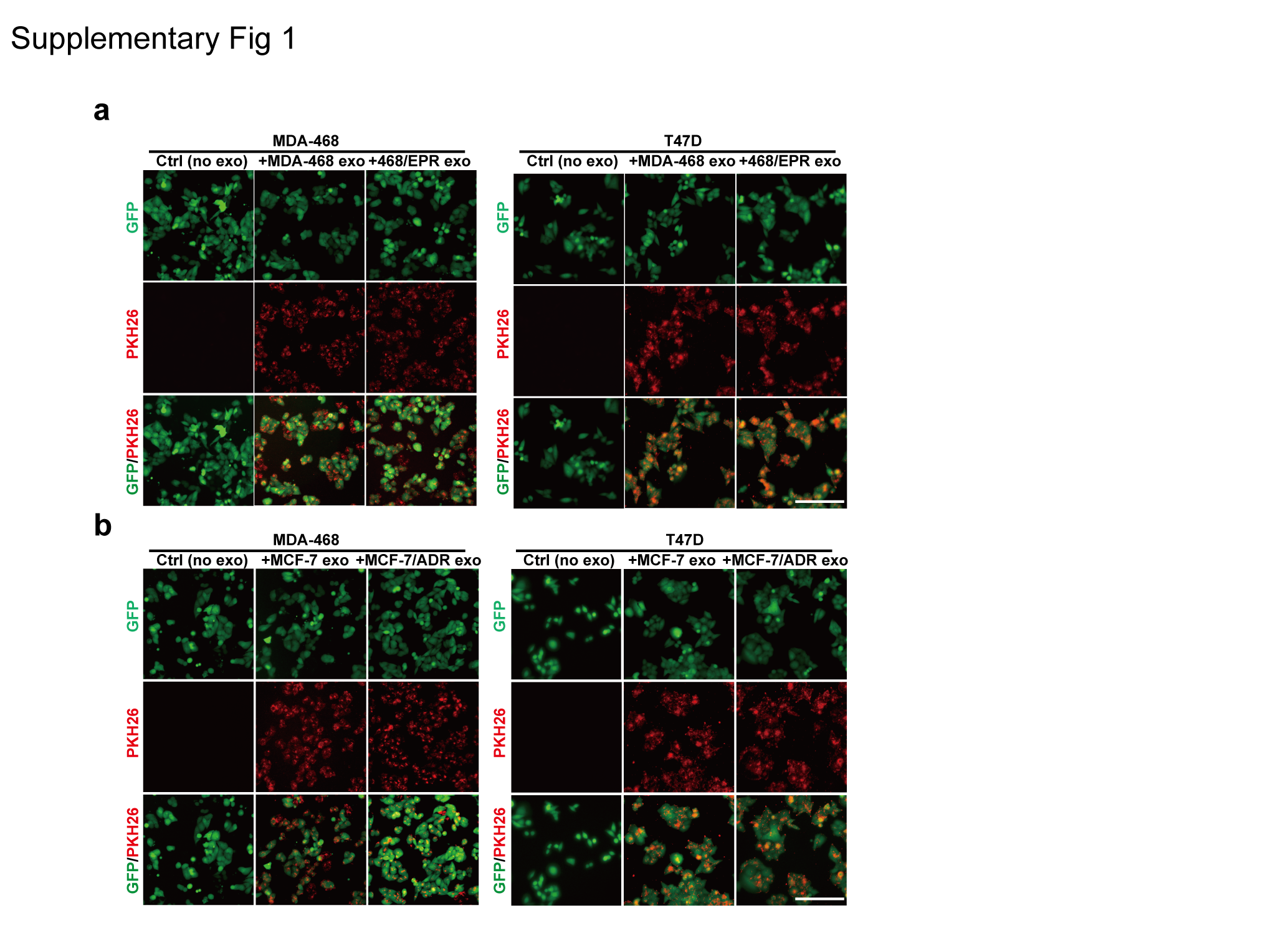

Supplement: Supplementary file 3 — Supplementary Fig 1 [file 41419_2021_3692_MOESM3_ESM.tif]

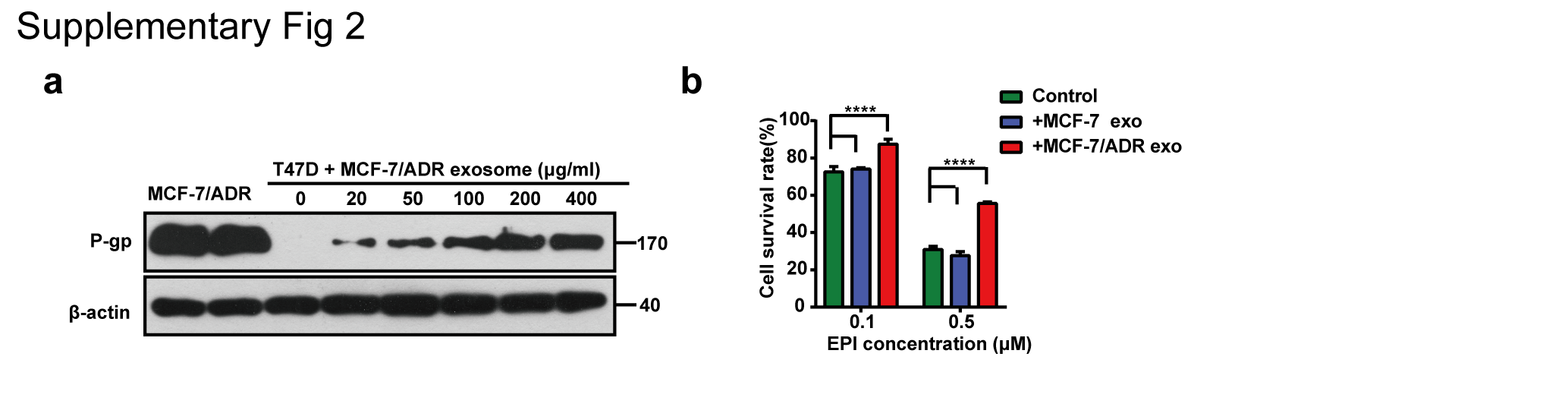

Supplement: Supplementary file 4 — Supplementary Fig 2 [file 41419_2021_3692_MOESM4_ESM.tif]

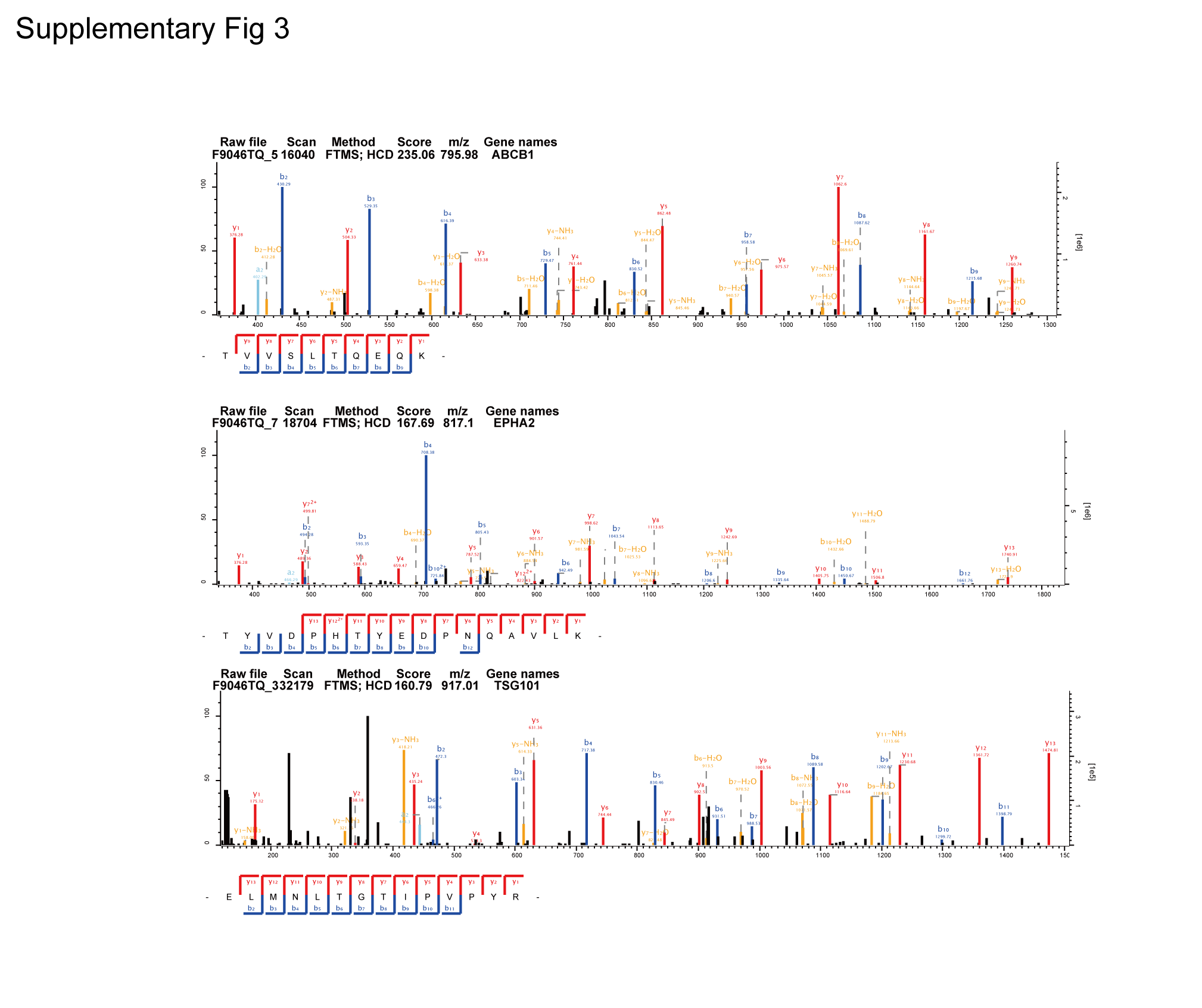

Supplement: Supplementary file 5 — Supplementary Fig 3 [file 41419_2021_3692_MOESM5_ESM.tif]

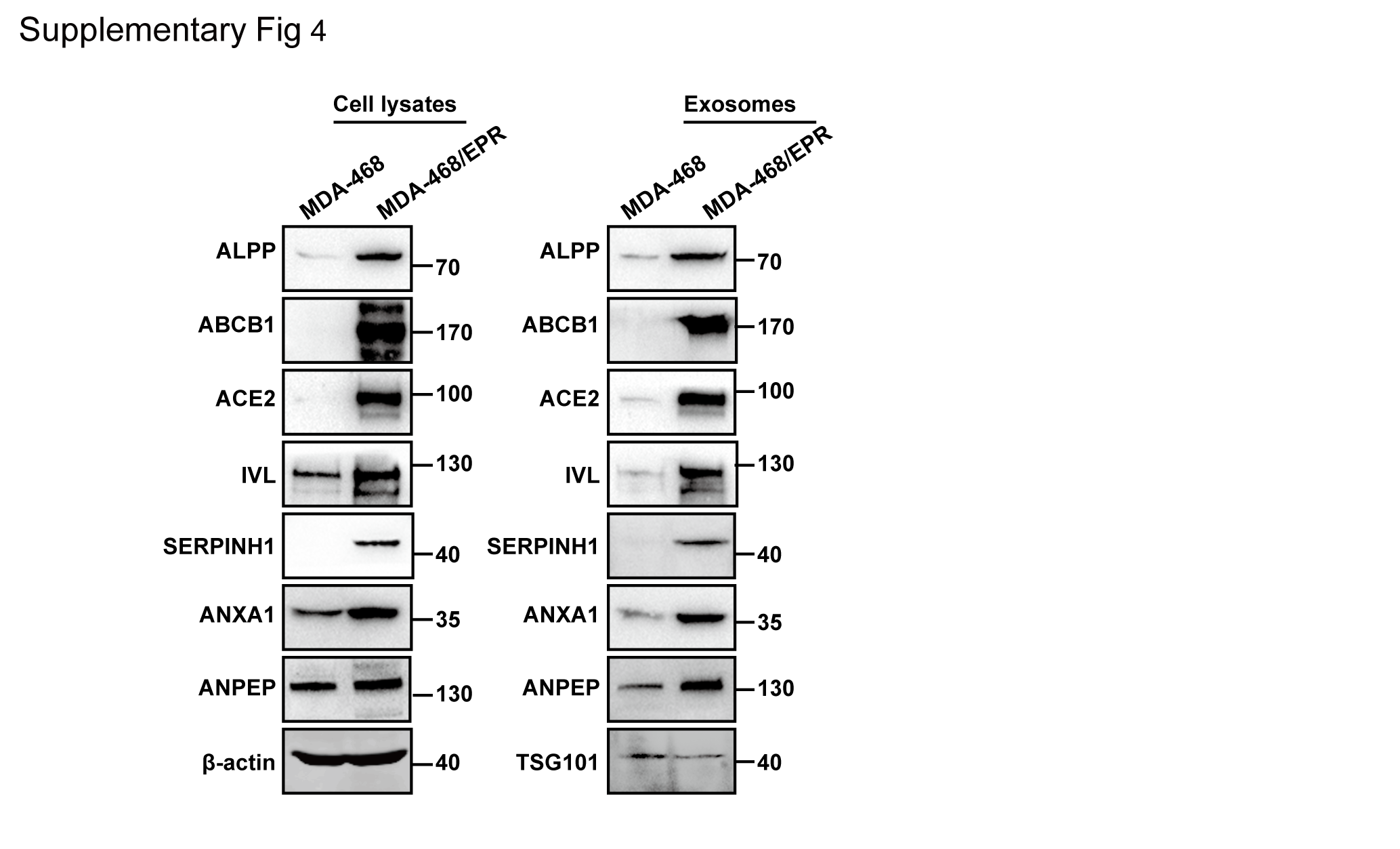

Supplement: Supplementary file 6 — Supplementary Fig 4 [file 41419_2021_3692_MOESM6_ESM.tif]

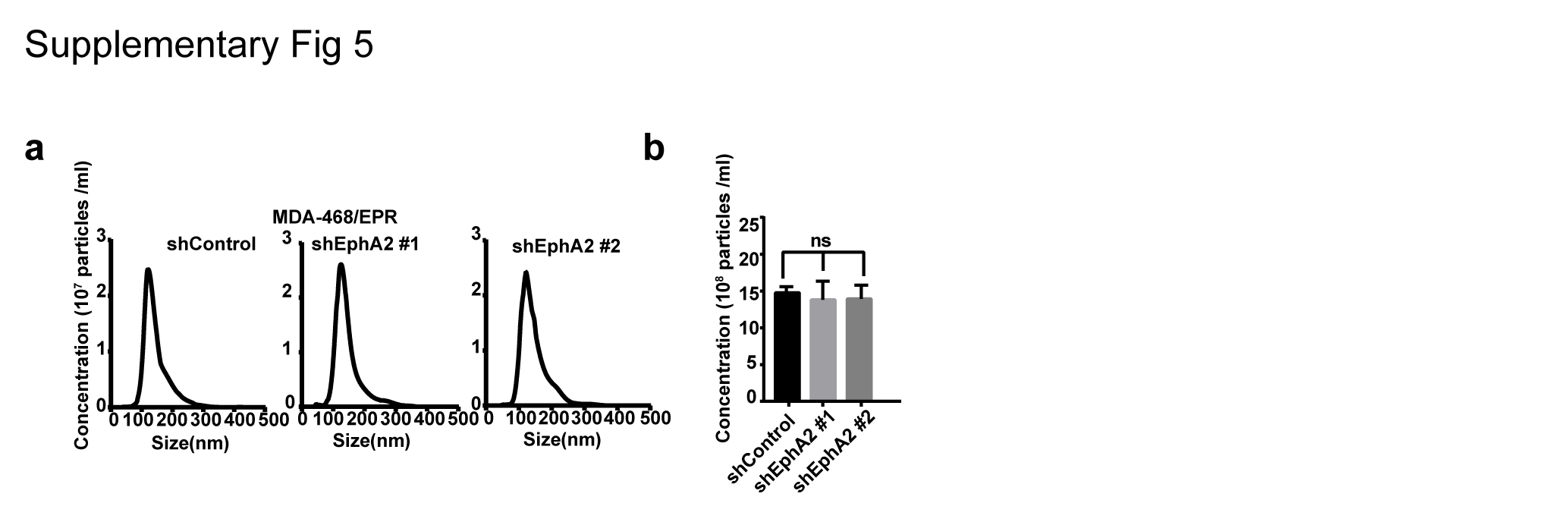

Supplement: Supplementary file 7 — Supplementary Fig 5 [file 41419_2021_3692_MOESM7_ESM.tif]

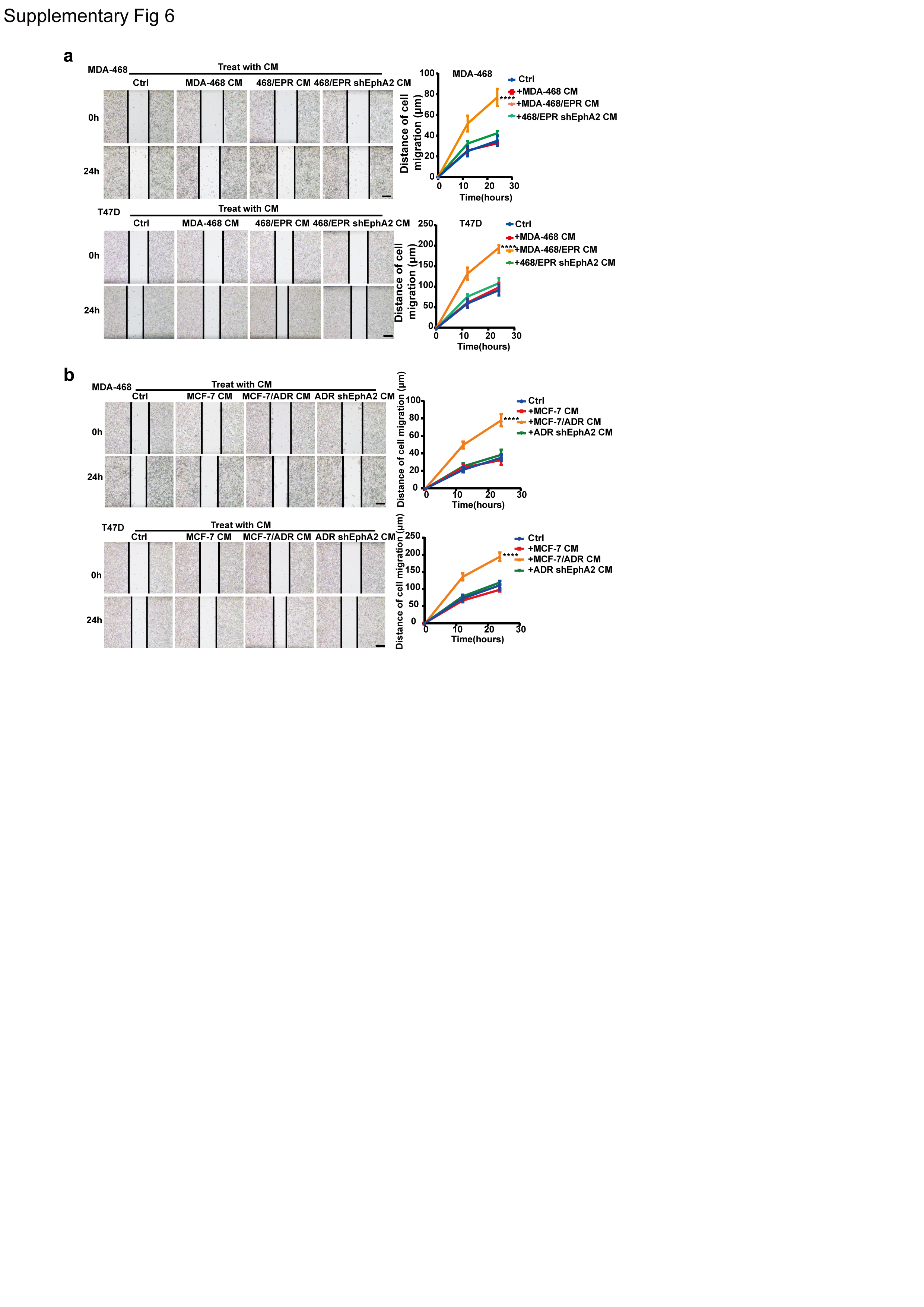

Supplement: Supplementary file 8 — Supplementary Fig 6 [file 41419_2021_3692_MOESM8_ESM.tif]

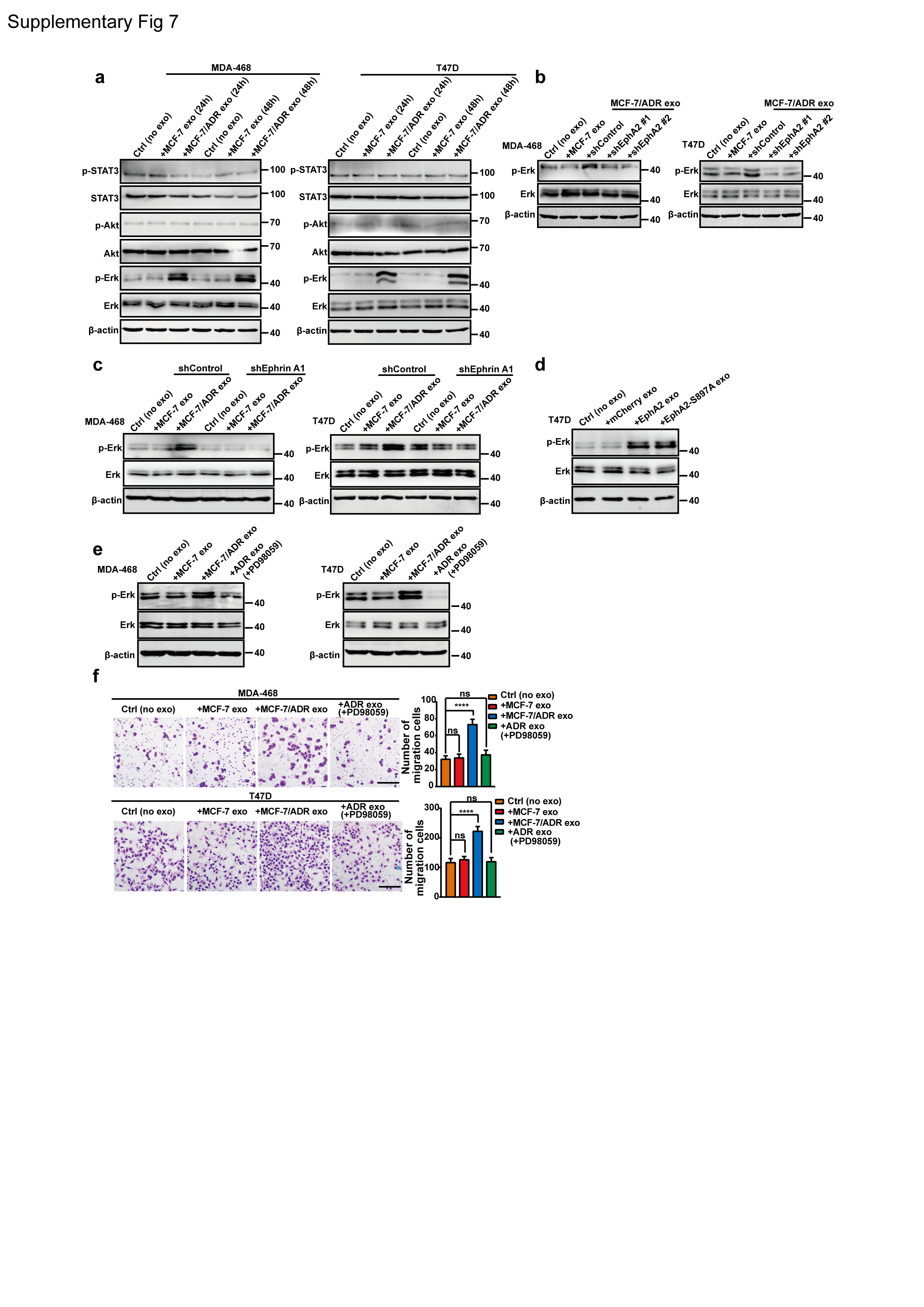

Supplement: Supplementary file 9 — Supplementary Fig 7 [file 41419_2021_3692_MOESM9_ESM.tif]

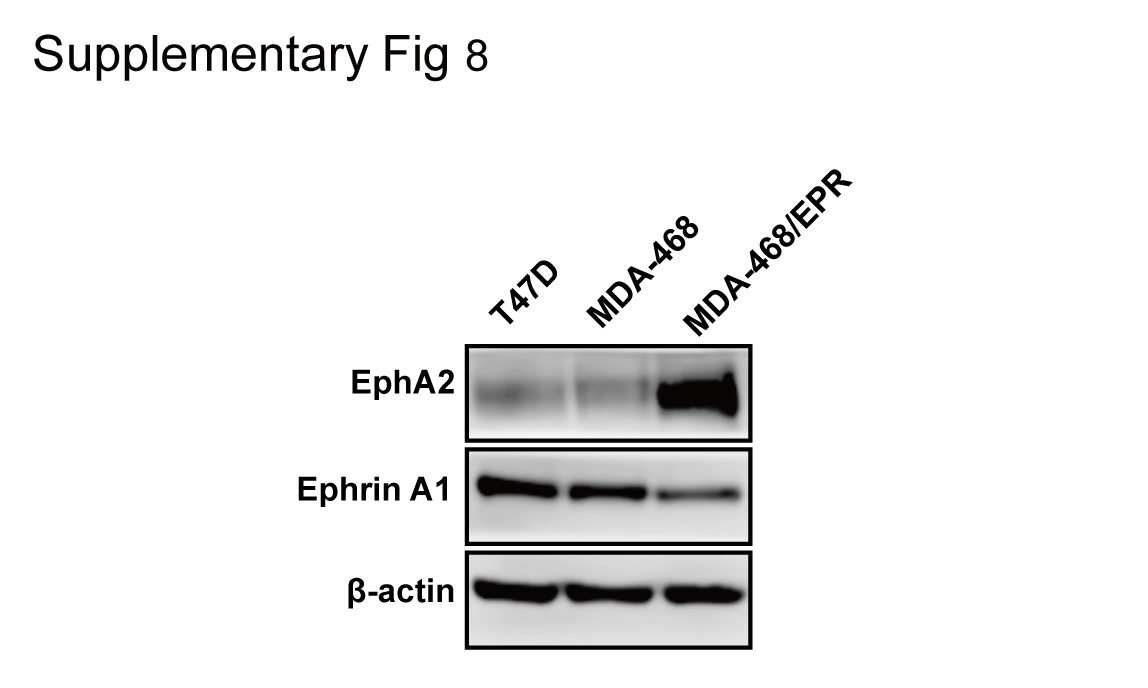

Supplement: Supplementary file 10 — Supplementary Fig 8 [file 41419_2021_3692_MOESM10_ESM.tif]
